# Comprehensive study of SNAREs involved in the post-Golgi transport in *Drosophila* photoreceptors

**DOI:** 10.3389/fcell.2024.1442192

**Published:** 2024-12-10

**Authors:** Yuka Ochi, Hitomi Yamashita, Shogo Sasaki, Takumi Ogawa, Yumi Yamada, Tatsuya Tago, Takunori Satoh, Akiko K. Satoh

**Affiliations:** Program of Life and Environmental Science, Graduate School of Integral Science for Life, Hiroshima University, Hiroshima, Japan

**Keywords:** *Drosophila*, photoreceptors, mosaic retinas, polarized transport, post-Golgi trafficking

## Abstract

Polarized transport is essential for the construction of multiple plasma membrane domains within cells. *Drosophila* photoreceptors serve as excellent model systems for studying the mechanisms of polarized transport. We conducted a comprehensive soluble N-ethylmaleimide-sensitive factor attachment protein receptor (SNARE) screening of the fly genome using RNAi knockdown and CRISPR/Cas9 somatic knockout combined with the CoinFLP system to identify SNAREs involved in post-Golgi trafficking. The results suggest that in post-Golgi transport, no SNARE is exclusively responsible for transport to a single specific plasma membrane domain. However, each SNARE shows some preference for certain membrane domains: the loss of nSyb, Ykt6, and Snap24/25 results in severe defects in rhabdomere transport, while the loss of Syx1A and Snap29 leads to significant impairments in basolateral transport. Together with the function of Syx1A, Snap25, and nSyb in the fusion of synaptic vesicles with the synaptic plasma membrane, these results suggest that SNAREs are not the sole determinants for vesicles to specify their target subdomains in the plasma membrane. Furthermore, rhodopsin transport to the rhabdomere requires two kinds of R-SNAREs, Ykt6 and nSyb, suggesting that multiple sets of post-Golgi SNAREs contribute in tandem or in cooperation, rather than in parallel.

## 1 Introduction

Many *in situ* cells, such as epithelial cells and neurons, show polarity and have more than two types of well-differentiated plasma membrane domains. Epithelial cells develop apical membranes for absorption or secretion and basolateral membranes for cell viability. Neurons contain dendrites for receiving signals and axons for transmitting signals to subsequent cells. These specific plasma membrane domains are formed and maintained by the accurate supply of specific membrane components from the trans-Golgi network (TGN) (Levic and Bagnat, 2022; Rodriguez-Boulan et al., 2005; Rodriguez-Boulan and Macara, 2014). These membrane flows are known as polarized transport and essential for human health because their impairment results in genetic disorders, including microvillus inclusion disease, polycystic kidney disease, and retinal degeneration (Charron et al., 2000; Cotton et al., 2013; El Ghouzzi and Boncompain, 2022; Hollingsworth and Gross, 2012; Müller et al., 2008; Ryan et al., 2010; Schneeberger et al., 2015; Vogel et al., 2017; Xiong and Bellen, 2013). Thus, it is important to understand the mechanisms of polarized transport.

Highly polarized *Drosophila* photoreceptors serve as excellent genetic model systems for studying the mechanisms of polarized transport (Hebbar et al., 2020; Schopf and Huber, 2017). By observing whole-mounted retinas using confocal microscopy, three distinct plasma membrane domains encompassing hundreds of photoreceptors were visualized simultaneously (Karagiosis and Ready, 2004). The first domain is the photoreceptive membrane domain, known as the rhabdomere, which forms at the center of the apical plasma membrane during pupal development. Proteins involved in phototransduction, such as the photosensitive molecule rhodopsin1 (Rh1), the Ca^2+^-permeable channel transient receptor potential, and chaoptin, a protein essential for rhabdomere architecture, localize specifically in the rhabdomeres (Hardie and Juusola, 2015). The second domain is the peripheral apical domain surrounding the rhabdomere, referred to as the stalk membrane, where the apical determinant Crumbs (Crb) is localized (Pellikka et al., 2002; Pocha et al., 2011). The third domain is the basolateral membrane, which is separated from the apical membrane by an adherens junction; Na^+^/K^+^-ATPase localizes to the basolateral membrane, similar to typical polarized epithelial cells (Yasuhara et al., 2000).

Several screening studies to identify proteins involved in polarized transport or recycling in fly retinas have been performed in the last decade (Laffafian and Tepass, 2019; Pocha et al., 2011; Satoh et al., 2013; Satoh et al., 2016a; Xiong et al., 2012; Yano et al., 2012; Feizy et al., 2024; Wang et al., 2014; Xiong et al., 2012; Zeger et al., 2024). These screenings, along with other studies, have revealed a number of proteins involved in transport to either the rhabdomere or basolateral membrane, as well as in recycling processes. Two sets of Rab proteins and their guanine nucleotide exchange factors, Rab6/Rich and Rab11/Pcs, regulate Rh1 transport to the rhabdomeres, and Rab10/Crag is required for basolateral transport, although Crag also works as Rab11GEF in adult photoreceptors under light conditions (Iwanami et al., 2016; Nakamura et al., 2020; Otsuka et al., 2019; Satoh et al., 2005; Tong et al., 2011; Xiong et al., 2012). The lack of motor proteins such as kinesin (Khc and Klc), dynein (Dhc64c), and MyoV (didum), or a subunit of tethering complex exocysts, causes Rh1-containing vesicles to accumulate in the cytoplasm (Beronja et al., 2005; Laffafian and Tepass, 2019; Li et al., 2007; Pocha et al., 2011; Wu et al., 2005; Yano et al., 2012). Conversely, the absence of adapter protein complex-1, clathrin, or glycosylphosphatidylinositol synthesis induces the mislocalization of the basolateral membrane protein Na^+^/K^+^-ATPase to the apical domains (Nakamura et al., 2020; Satoh et al., 2013). It is not known whether there is a specific transport pathway to the stalk membrane. To date, no factor involved in such a pathway has been identified. In addition, the stalk/rhabdomere boundary lacks an obvious physical structure separating these subdomains, such as an adherens junction separating the stalk and basolateral subdomains (Schopf and Huber, 2017). These results suggest that proteins destined for the apical plasma membrane are transported by the same pathway and then segregate into the stalk and rhabdomere subdomains by soluble N-ethylmaleimide-sensitive factor attachment protein receptor (SNARE)-independent mechanisms.

SNARE proteins are indispensable components for membrane fusion (Grissom et al., 2020; Hong and Lev, 2014; Jahn et al., 2024; Stanton and Hughson, 2023). They are classified as R-SNAREs and Q-SNAREs based on the presence of conserved arginine or glutamine in the SNARE motif. Q-SNAREs are further subdivided into Qa-, Qb-, and Qc-SNAREs based on their homology to syntaxin and SNAP25. In *Drosophila* photoreceptors, phenotypes of loss-of-function of six SNAREs have been reported: Sec22, an R-SNARE that is essential for endoplasmic reticulum (ER) morphology and photoreceptor morphogenesis; Gos28, a Qb-SNARE that mediates intra-Golgi transport of Rh1 and is required for photoreceptor survival; Syx5, a Qa-SNARE that regulates ER-to-Golgi transport; Syx7, a Qa-SNARE that is required for the degradation of large amounts of endocytosed apical components; and R-SNAREs nSyb and Syb that are required for the post-Golgi transport of Rh1, which was recently reported (Laffafian and Tepass, 2019; Rosenbaum et al., 2014; Satoh et al., 2016b; Yamashita et al., 2022; Zhao et al., 2015). These studies highlight the functions of SNAREs in photoreceptor morphogenesis and survival in flies; however, SNAREs involved in polarized transport have not been identified.

In this study, we used RNAi knockdown and a somatic knockout (KO) approach to search for SNARE proteins involved in post-Golgi transport to the rhabdomere, stalk, or basolateral membrane. Surprisingly, in the post-Golgi transport, none of the SNAREs are exclusively involved in the transport to a specific destination on the plasma membrane domains, although each SNARE shows some preference for certain membrane domains. In particular, Syx1A, Snap24, and Snap25 appear to be required for all plasma membrane domains.

## 2 Materials and methods

### 2.1 Construction of the plasmids and transgenic flies

Details of the DNA constructs used in this paper are given in Supplemental Materials and Methods. Molecular cloning was performed according to standard protocols. In brief, PCRs were performed using a KOD One PCR Master Mix (TOYOBO Osaka, Japan), ligations were performed using Ligation high Ver.2 (TOYOBO), and Gibson assemblies were performed using a NEBuilder HiFi DNA Assembly Master Mix (New England Biolabs, Ipswich, MA, United States). The ligated or assembled DNAs were chemically transformed into *E. coli* OmniMAX 2T1R (Thermo Fisher Scientific, Waltham, MA, United States). Sequence files of plasmids constructed for this paper are available in Dryad (https://doi.org/10.5061/dryad.wwpzgmssx) and given in Supplementary Table S3. Primers used for PCR and adapters for fill-in are given in Supplementary Table S4.

### 2.2 *Drosophila* stocks and genetics

Flies were grown at 20°C–25°C on standard cornmeal–glucose–agar–yeast food. The following fly stocks were used: Rh1-Gal4 (from Dr. Hama, Kyoto Sangyo University) and vas-Cas9 (stock number 51325, Bloomington *Drosophila* Stock Center, Bloomington, IN, United States: BL51325). The SNARE RNAi lines used in this study are given in Supplementary Table S1. X chromosomes of SNARE RNAi lines obtained from the stock centers were replaced with those carrying eyeless-Flippase (eyFLP) and UAS-Dicer. These lines were crossed with CoinFLP-ActGal4 (BL58751) (Bosch et al., 2015) or CoinFLP-longGMR-Gal4, which were prepared in this work (see below). The SNARE gRNA lines used in this study are given in Supplementary Table S2. These lines were crossed with CoinFLP-longGMR-FLAG::Cas9-H2B::NG or CoinFLP-Act5C-FLAG::Cas9-H2B::GFP, which were prepared in this work (see below). To obtain Syx7 null mosaic eyes, w*;; FRT80B, Syx7^1^/TM6B (BL52000) and w*;; FRT80B, Syx7^4^/T (2;3) TSTL, CyO; TM6B, Tb^1^ (BL51644) were crossed to y w ey-FLP;; P3RFP FRT80B (Satoh et al., 2013). To obtain Syx6 null mosaic eyes, y w ey-FLP; FRT42D Syx6^Δ5^/CyO GFP prepared in this work (see below) were crossed to y w ey-FLP; FRT42D P3RFP (Satoh et al., 2013). To obtain Syx13 null mosaic eyes, y w ey-FLP; Syx13^Δ11^ FRT80B/TM6B prepared in this work (see below) were crossed to y w ey-FLP;; P3RFP FRT80B (Satoh et al., 2013).

### 2.3 Generation of transgenic flies

A Phi31-based vector, pCFD4w-Syx6, was injected into the embryo of a phi31 host line (y[1] M{vas-int.Dm}ZH-2A w[*]; M{3xP3-RFP.attP'}ZH-68E, BL24485) to obtain gRNA-Syx6. P-element-based vectors were co-injected with a helper plasmid pUC hsPI{delta2-3} (DGRC Stock 1001; https://dgrc.bio.indiana.edu//stock/1001; RRID:DGRC_1001) into the embryo of a host line, w^1118^. The plasmid of pP-3xlongGMR-coinFLP-HFCas9-H2B-2xNG was injected into the w^1118^ embryo by BestGene (CA, United States).

### 2.4 Generation of null alleles for Syx13 and Syx6 genes by the CRISPR/Cas9 system

The male flies carrying Syx13-gRNA on second (BL84021), y sc v sev/Y; P{TKO.GS04362}attP40, were crossed to females carrying vas-Cas9 and FRT80B, y w M{vas-Cas9}ZH-2A;; Syx13^wt^ FRT80B. Each male offspring, y w M{vas-Cas9}ZH-2A/ Y; P{TKO.GS04362}attP40/+; Syx13^KO^ FRT80B, was individually crossed with females, y w eyFLP;; P3RFP FRT80B. Each male offspring with RFP-mosaic eyes, y w eyFLP/Y;; Syx13^KO^ FRT80B/P3RFP FRT80B, was crossed with females, y w eyFLP;; Dr P{hs-hid}/TM6B GFP, to establish stock lines, and the males were lysed for genotyping. The genomic region, including the target site of CRISPR-Cas9, was amplified with Syx13-GF1 and Syx13-GR1 and sequenced (Supplementary Tables S3, S4). Syx13^Δ11^ carrying 11 base deletions (GCTTCAGTCCG) was identified as the null allele.

The males flies carrying Syx6-gRNA on third, w/Y;; Syx6-gRNA/TM6B, were crossed to females carrying vas-Cas9 and FRT42D, y w M{vas-Cas9}ZH-2A; Syx6^wt^ FRT42D. Each male offspring, y w M{vas-Cas9}ZH-2A/Y; Syx6^KO^ FRT42D/+; Syx6-gRNA/+, was individually crossed with females, y w eyFLP; P3RFP FRT42D. Each male offspring with RFP-mosaic eyes, y w eyFLP/Y; Syx6^KO^ FRT42D/P3RFP FRT42D, was crossed with females, y w eyFLP; Sp P{hs-hid}/CyO GFP, to establish stock lines, and the males were lysed for genotyping. The genomic region, including the target site of CRISPR-Cas9, was amplified with Syx6-GF3 and Syx6-GR3 and then sequenced (Supplementary Tables S3, S4). Syx6^Δ5^ carrying five base deletions (TGTCG) was identified as the null allele.

### 2.5 Immunohistochemistry

Fixation and staining methods were performed as described previously (Otsuka et al., 2019; Satoh and Ready, 2005). The primary antisera were as follows: rabbit anti-Rh1 (1:1,000) (Satoh et al., 2005), mouse monoclonal anti-Na^+^/K^+^-ATPase alpha subunit (α5: 1:500 ascite; Developmental Studies Hybridoma Bank (DSHB) Iowa City, IA, United States), mouse anti-Na^+^/K^+^-ATPase beta subunit (Nrv) (1:10) (DSHB, IA, United States), mouse anti-Eys (1:10) (DSHB, IA, United States), rabbit anti-Myc (1:300) (Medical and Biological Laboratories, Nagoya, Japan; No. 562), rabbit anti-HA (1:300; cat. no. 561, Medical and Biological Laboratories, Nagoya, Japan), mouse anti-Syx1A (1:15) (DSHB, IA, United States), rabbit anti-TRP (1:1000) (Dr. Montell, University of California, Santa Barbara, United States), rat anti-Crb (1:300) (Dr. Tepass, University of Toronto, Canada), rabbit anti-Snap29 (1:300; provided by Dr. Paden, University of Sheffield, United Kingdom), rabbit anti-Sec22 (1:300) (Dr. Paden, University of Sheffield, United Kingdom), guinea pig anti-Rab6 (1:300) (Iwanami et al., 2016), and rat anti-Rab11 (1:300) (Otsuka et al., 2019). The secondary antibodies were anti-mouse, anti-rabbit, and/or anti-rat antibodies labeled with Alexa Fluor 488, 568, and 647, respectively (1:300; Life Technologies, Carlsbad, CA, United States). Phalloidin-conjugated Alexa Fluor 568 (1:100; Life Technologies, Carlsbad, CA, United States) was used for F-actin staining. Images of samples were recorded using a FV3000 confocal microscope (UPLXAPO60XO 1.30 NA and UPlanSApo 60 × S2 1.42 NA objective lens; Olympus, Tokyo, Japan). To minimize bleed-through, each signal in double- or triple-stained samples was imaged sequentially. The images were processed in accordance with the Guidelines for Proper Digital Image Handling using ImageJ and/or Affinity Photo (Serif Europe Ltd., West Bridgford, Nottinghamshire, United Kingdom) (Schindelin et al., 2012). For the quantification of the intensity of Rh1 and Na^+^/K^+^-ATPase staining in the photoreceptor cytoplasm, we used more than three mosaic retinas with more than eight wild-type and more than six mutant photoreceptors in each retina. For the quantification of Rh1, the area of cytoplasm or whole cells and their staining intensities were measured, and for the quantification of Na^+^/K^+^-ATPase-α and Nrv, the staining intensities of the cytoplasm were measured using Fiji (Schindelin et al., 2012).

### 2.6 Electron microscopy

Electron microscopy was performed as described previously (Satoh et al., 1997). The samples were observed under a JEM-1400 and JEM-1400Plus electron microscope (JEOL, Tokyo, Japan), and montages were prepared using a CCD camera system (JEOL). The phenotypes were investigated using the section with the depth where a couple of photoreceptor nuclei within the ommatidia were observed.

## 3 Results

### 3.1 Three-step RNAi mosaic eye screening

Mosaic retinas, consisting of both wild-type and mutant cells, provide a powerful tool for assessing the phenotype of mutant photoreceptors because they can be compared side-by-side with wild-type cells. The previously reported CoinFLP system (Bosch et al., 2015) enables mosaic RNAi knockdown screening by automatically generating a reliable ratio of mutant-to-wild-type tissues in a developmentally controlled manner. In brief, in the CoinFLP system, strong and constitutive expression of the Gal4 trans-activator from the Act5C promoter is blocked by the “transcriptional stop cassette” until it is unlocked by recombination between two canonical flippase recognition target (FRT) sites mediated by FLP recombinase. Instead, in approximately half of the cells, the recombination between two FRT3 sites anchors a “transcriptional stop cassette” upstream of Gal4, resulting in the permanent inactivation of Gal4 expression. Combining CoinFLP with any FLP lines that are expressed in the tissues and the timing of interest, in our case, retinas, allows the generation of mosaic tissues consisting of mutant and wild-type cells. In this study, we used the promoter of the *eyeless* gene, the master regulator of the eye, to express the FLP recombinase at an early stage of eye imaginal disk development. We named this configuration eyeless–CoinFLP–Act5C–Gal4 (Bosch et al., 2015) and used it as the first step on the screen.

**FIGURE 1:**
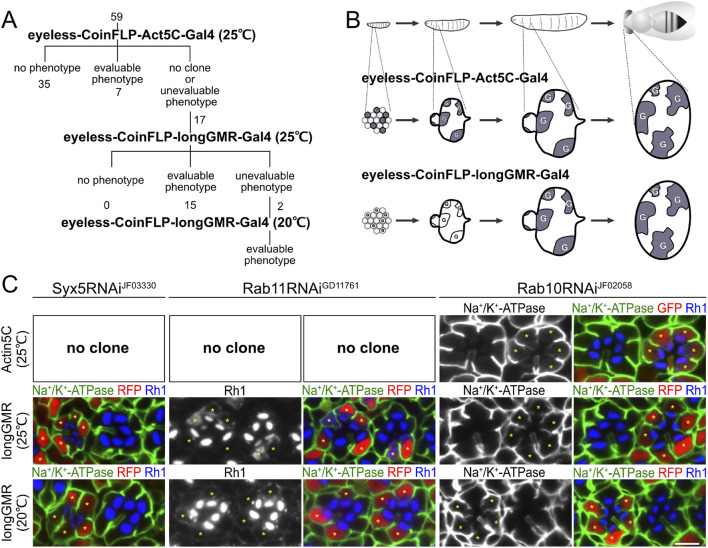
Three-step RNAi screening of SNAREs involved in polarized transport in *Drosophila* photoreceptors. **(A)** Schematic diagram of RNAi screening of SNAREs involved in polarized transport. **(B)** Schematic diagram of the Gal4 expression pattern in developing eyes by eyeless–CoinFLP–Act5C–Gal4 and eyeless–CoinFLP–longGMR–Gal4. Alteration of the original schematic diagram in the study by Bosch et al. (2015). **(C)** Immunostaining of Syx5RNAi^JF03330^-, Rab11RNAi^GD11761^-, or Rab10RNAi^JF02058^-expressing retina by eyeless–CoinFLP–Act5C–Gal4 and eyeless–CoinFLP–longGMR–Gal4 using anti-Na^+^/K^+^-ATPase-α (green) and anti-Rh1 (blue) antibodies. GFP or RFP (red) and asterisks represent the cells expressing RNAi constructs. Scale bar: 5 μm **(C)**.

The expression of 59 types of RNAi constructs driven by eyeless–CoinFLP–Act5C–Gal4 resulted in 7 lines with well-grown Gal4-positive clones showing obvious phenotypes, 35 lines without any phenotype, and 17 lines with no Gal4-positive clones or cells with phenotypes too severe for analysis (Figure 1A). We hypothesized that the loss of Gal4-positive cells was the result of a lack of clonal growth or cell survival caused by the constant expression of the RNAi construct.

To evaluate the effects of the 17 RNAi lines, we attempted to delay the onset of Gal4 expression in the second step of screening. Both the GMR and longGMR promoters have been used to induce expression in all developing retinal cells, starting in the third-instar larvae, i.e., posterior to the morphogenetic furrow (Wernet et al., 2003). The longGMR promoter provides milder but more photoreceptor-specific expression than the GMR promoter (Wernet et al., 2003). Thus, we generated a transgenic fly with the CoinFLP–Gal4 construct under the control of the longGMR promoter by replacing the Act5C promoter. On combining it with an eyeless-FLP, we referred to this configuration as eyeless–CoinFLP–longGMR–Gal4 (Figure 1B). We crossed them with 17 lines of RNAi constructs, which failed to obtain evaluable clones when crossed with the eyeless–CoinFLP–Act5C–Gal4 system. Gal4-positive clones with milder and more evaluable phenotypes were obtained from 15 of the 17 RNAi lines. However, two of these lines exhibited phenotypes that were too severe for evaluation. As the third step in the screen, we reared the flies at lower temperatures to reduce the effect of RNAi because Gal4 activity is temperature-dependent. Flies expressing these two RNAi constructs in eyeless–CoinFLP–longGMR–Gal4 were maintained at low temperatures, resulting in the eventual acquisition of evaluable clones.

### 3.2 Validation of the three-step RNAi-mosaic eye screening

To validate the results of the three-step RNAi screening, we first examined the phenotypes induced by *Syx5*, *Rab10*, or *Rab11* RNAi constructs under the three conditions and compared them with the phenotypes previously reported for the loss of the *Syx5*, *Rab10*, and *Rab11* genes (Figure 1C). Syx5 is involved in early Golgi trafficking in fly photoreceptor cells; in homozygous clones of hypomorphic allele *Syx5* ^*EP2313*^, ommatidia are smaller than those in wild-type clones, and the photoreceptors have less Rh1 localizing only in the rhabdomeres (Satoh et al., 2016b). The RNAi construct targeting *Syx5*, Syx5RNAi^JF03330^, failed to produce any clones when driven by eyeless–CoinFLP–Act5C–Gal4 but produced reasonably grown clones when driven by eyeless–CoinFLP–longGMR–Gal4. In such clones, the ommatidia are small, and the photoreceptors have less Rh1, which is limited only to the rhabdomeres and replicates the phenotypes of the homozygous *Syx5* ^*EP2313*^ clones. We previously reported that Rab11 is involved in post-Golgi transport to the rhabdomeres; in the photoreceptor homozygous null mutant *Rab11* ^*EP3017*^ and photoreceptors expressing the dominant-negative mutant Rab11^N124I^, rhabdomeres are small, and Rh1 is not concentrated in the rhabdomeres but accumulates in the cytoplasm (Satoh et al., 2005). In this study, Rab11RNAi^GD11761^ did not produce any clones by eyeless–CoinFLP–Act5C–Gal4 but produced them by eyeless–CoinFLP–longGMR–Gal4. Rhabdomeres were small, and Rh1 accumulated in the cytoplasm of clones expressing Rab11RNAi^GD11761^, which perfectly phenocopied *Rab11* ^*EP3017*^ homozygous cells and photoreceptors expressing dominant-negative Rab11^N124I^. In Rab11RNAi^GD11761^ clones generated by eyeless–CoinFLP–longGMR–Gal4 at lower temperatures, the rhabdomeres grew to a normal size, and only low Rh1 accumulation was detected. Thus, the strength of the phenotypes can be manipulated by the type of CoinFLP–Gal4 driver and temperature. In contrast, the phenotype of the Rab10RNAi^JF02058^ clone was not significantly attenuated by the type of CoinFLP–Gal4 driver or ambient temperature. Rab10 is required for the basolateral transport of Na^+^/K^+^-ATPase in fly photoreceptors, and dominant-negative Rab10S23N expression or Rab10 deficiency induces the mislocalization of Na^+^/K^+^-ATPase to the stalk membrane (Nakamura et al., 2020; Ochi et al., 2022). In Rab10RNAi^JF02058^ clones generated with either eyeless–CoinFLP–Act5C–Gal4 or eyeless–CoinFLP–longGMR–Gal4, Na^+^/K^+^-ATPase was mislocalized to the stalk membrane, even when grown at a lower temperature (20°C). These results indicate that the three-step RNAi mosaic screening scheme can mitigate the difficulties arising from the unpredictable efficacy of RNAi, thus providing a powerful method for screening genes involved in biosynthetic transport pathways in *Drosophila* photoreceptors.

### 3.3 Classification of SNARE proteins by knockdown phenotypes

**FIGURE 2:**
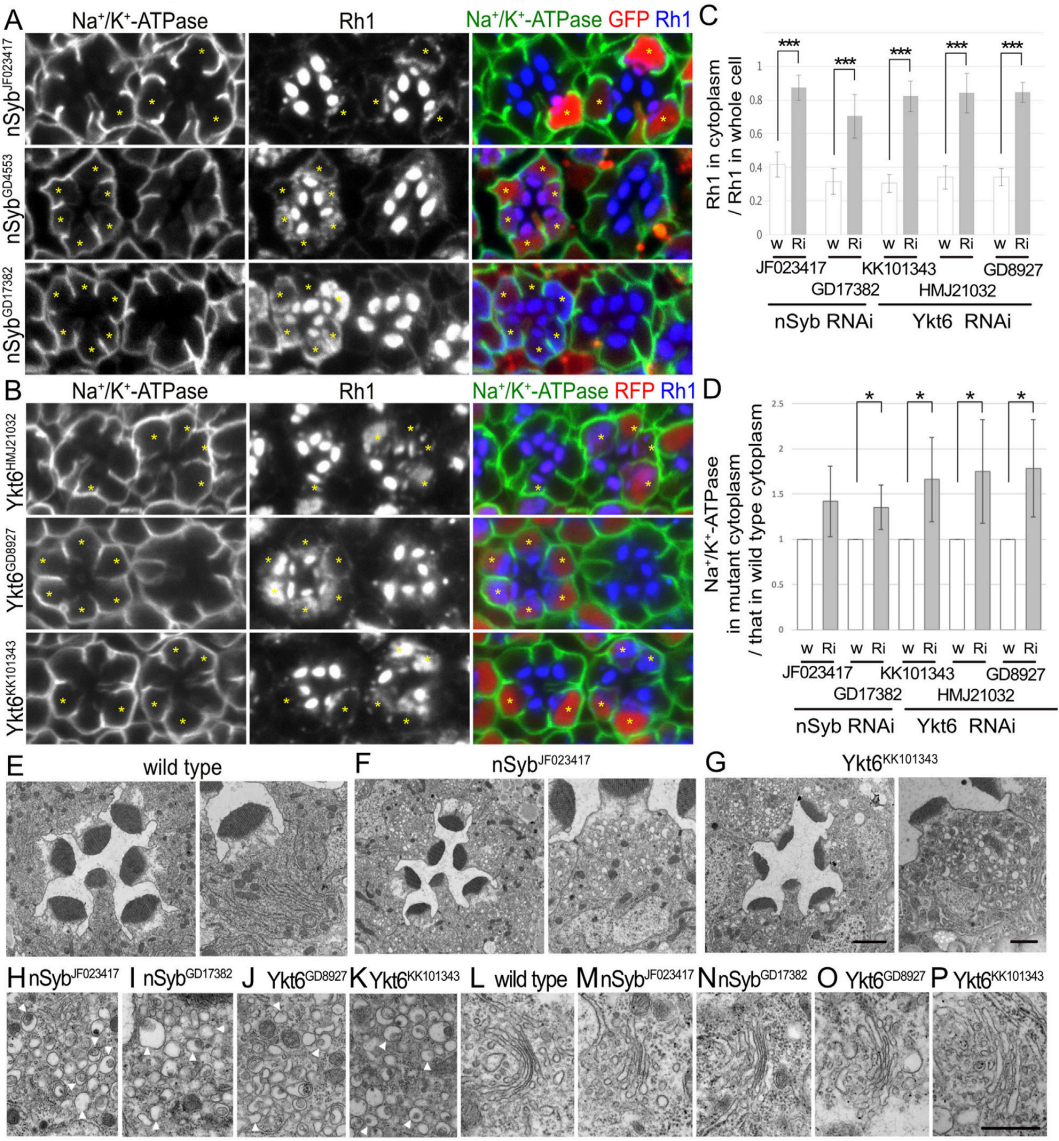
nSyb and Ykt6 are required for the post-Golgi transport toward the rhabdomeres. **(A)** Immunostaining of nSybRNAi^JF023417^-, nSybRNAi^GD4553^-, or nSybRNAi^GD17382^-expressing retina by eyeless–CoinFLP–Act5C–Gal4 using anti-Na^+^/K^+^-ATPase-α (green) and anti-Rh1 (blue) antibodies. GFP (red) and asterisks represent the cells expressing RNAi constructs. **(B)** Immunostaining of Ykt6RNAi^HMJ21032^-, Ykt6RNAi^GD8927^-, or Ykt6RNAi^KK101343^-expressing retina by eyeless–CoinFLP–longGMR–Gal4 using anti-Na^+^/K^+^-ATPase-α (green) and anti-Rh1 (blue) antibodies. RFP (red) and asterisks represent the cells expressing RNAi constructs. **(C)** The ratio of integrated fluorescence density for Rh1 staining of the cytoplasm compared with that of whole cells was plotted. White bars indicate wild-type cells (w), and gray bars indicate the cells expressing RNAi constructs (Ri). Error bars indicate the SD with five retinas. Significance according to two-tailed unpaired Student’s *t-*test: ***p < 0.001. **(D)** The ratio of integrated fluorescence density for Na^+^/K^+^-ATPase-α staining of the cytoplasm in the cells expressing RNAi constructs compared with that in wild-type cells was plotted. White bars indicate wild-type cells (w), and gray bars indicate the cells expressing RNAi constructs (Ri). Error bars indicate the SD with four retinas. Significance according to two-tailed unpaired Student’s *t-*test: *p < 0.05. **(E–G)** Electron micrographs of the wild-type **(D)**, nSybRNAi^JF023417^ **(E)**, Ykt6RNAi^GD8927^ **(F)**, or Ykt6RNAi^KK101343^ **(G)** ommatidium (left) and photoreceptor (right). **(H–K)** Electron micrographs of the vesicles accumulated in the cytoplasm of the photoreceptors expressing nSybRNAi^JF023417^ **(H)**, nSybRNAi^GD17382^ **(I)**, Ykt6RNAi^GD8927^ **(J)**, or Ykt6RNAi^KK101343^ **(K)**. Arrowheads indicate unusual irregular-shaped vesicles. **(L)** Electron micrographs of the Golgi stacks of wild-type photoreceptors. **(M–P)** Electron micrographs of the Golgi stacks of the photoreceptors expressing nSybRNAi^JF023417^ **(M)**, SybRNAi^GD17382^ **(L, N)**, Ykt6RNAi^GD8927^ **(O)**, or Ykt6RNAi^KK101343^ **(P)**. Scale bar: 5 μm **(A, B)**, 2 μm [**(E–G)** left], 1 μm [**(E–G)** right], and 500 nm **(H–P)**.

**FIGURE 3:**
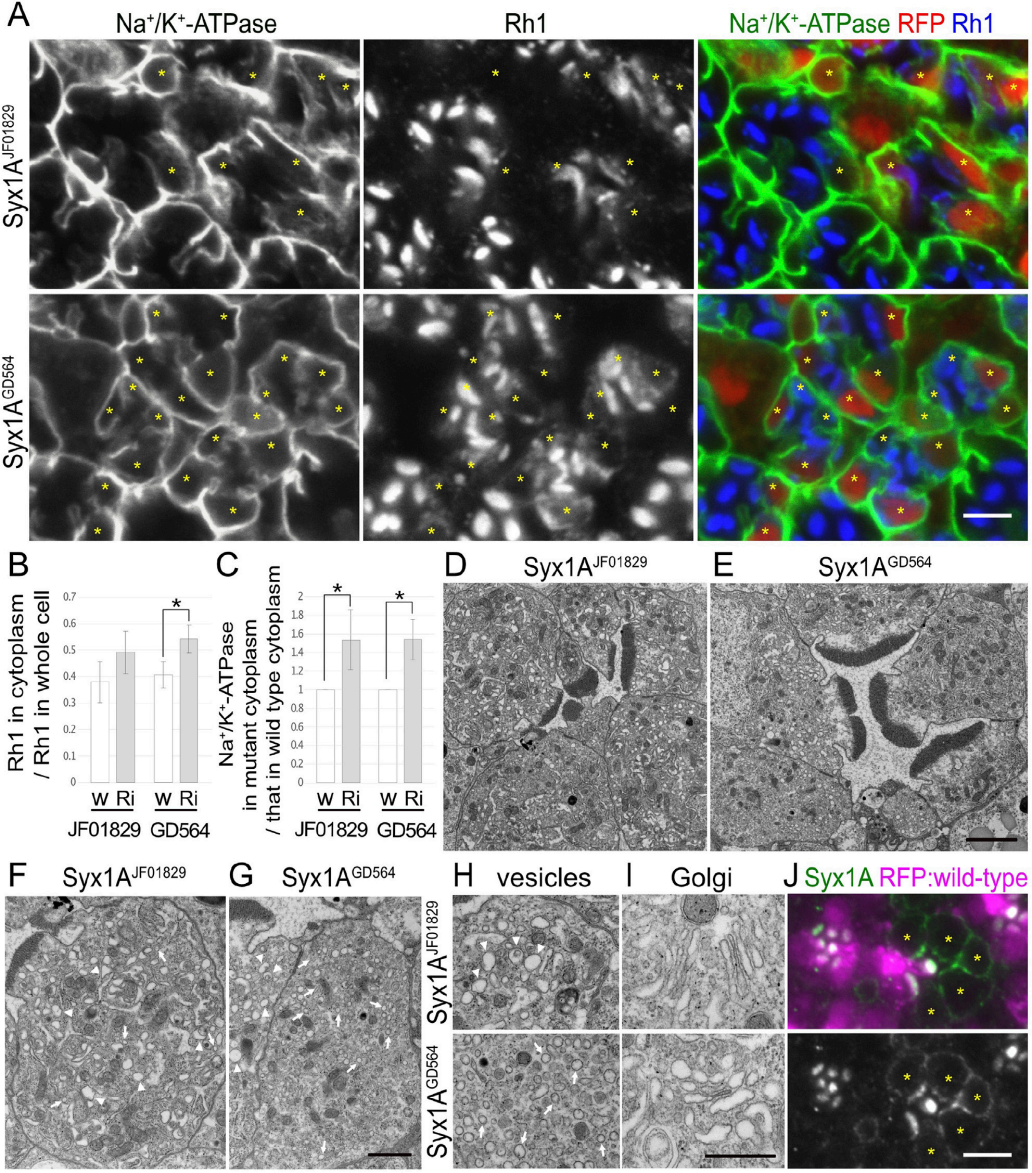
Syx1A is required for the polarized transports toward both the rhabdomeres and basolateral membrane. **(A)** Immunostaining of Syx1ARNAi^JF01829^- or Syx1ARNAi^GD564^-expressing retina by eyeless–CoinFLP–longGMR–Gal4 using anti-Na^+^/K^+^-ATPase-α (green) and anti-Rh1 (blue) antibodies. RFP (red) and asterisks represent the cells expressing RNAi constructs. **(B)** The ratio of integrated fluorescence density for Rh1 staining of the cytoplasm compared with that of whole cells was plotted. White bars indicate wild-type cells (w), and gray bars indicate the cells expressing RNAi constructs (Ri). Error bars indicate the SD with four retinas. Significance according to two-tailed unpaired Student’s *t-*test: *p < 0.05. **(C)** The ratio of integrated fluorescence density for Na^+^/K^+^-ATPase-α staining of the cytoplasm in the cells expressing RNAi constructs compared with that in wild-type cells was plotted. White bars indicate wild-type cells (w), and gray bars indicate the cells expressing RNAi constructs (Ri). Error bars indicate the SD with four retinas. Significance according to two-tailed unpaired Student’s *t-*test: *p < 0.05. **(D, E)** Electron micrographs of the ommatidium expressing Syx1ARNAi^JF01829^ **(D)** or Syx1ARNAi^GD564^ **(E)**. **(F, G)** Electron micrographs of the photoreceptor expressing Syx1ARNAi^JF01829^ **(F)** or Syx1ARNAi^GD564^ **(G)**. Arrowheads and arrows indicate unusual irregular-shaped vesicles and normal vesicles, respectively. **(H)** Electron micrographs of the vesicles accumulating in the cytoplasm in the photoreceptors expressing Syx1ARNAi^JF01829^ (upper) and Syx1ARNAi^GD564^ (lower). Arrowheads and arrows indicate unusual irregular-shaped vesicles and normal vesicles, respectively. **(I)** Electron micrographs of Golgi stacks in the photoreceptors expressing Syx1ARNAi^JF01829^ (upper) and Syx1ARNAi^GD564^ (lower). **(J)** Immunostaining of *EMC3* ^*Δ4*^ mosaic retina using anti-Syx1A antibody (green). RFP (magenta) and asterisks represent the wild-type cells and mutant cells, respectively. Scale bar: 5 μm **(A)**, 2 μm **(D, E)**, 1 μm **(F, G)**, 500 nm **(H, I)**, and 5 μm **(J)**.

**FIGURE 4:**
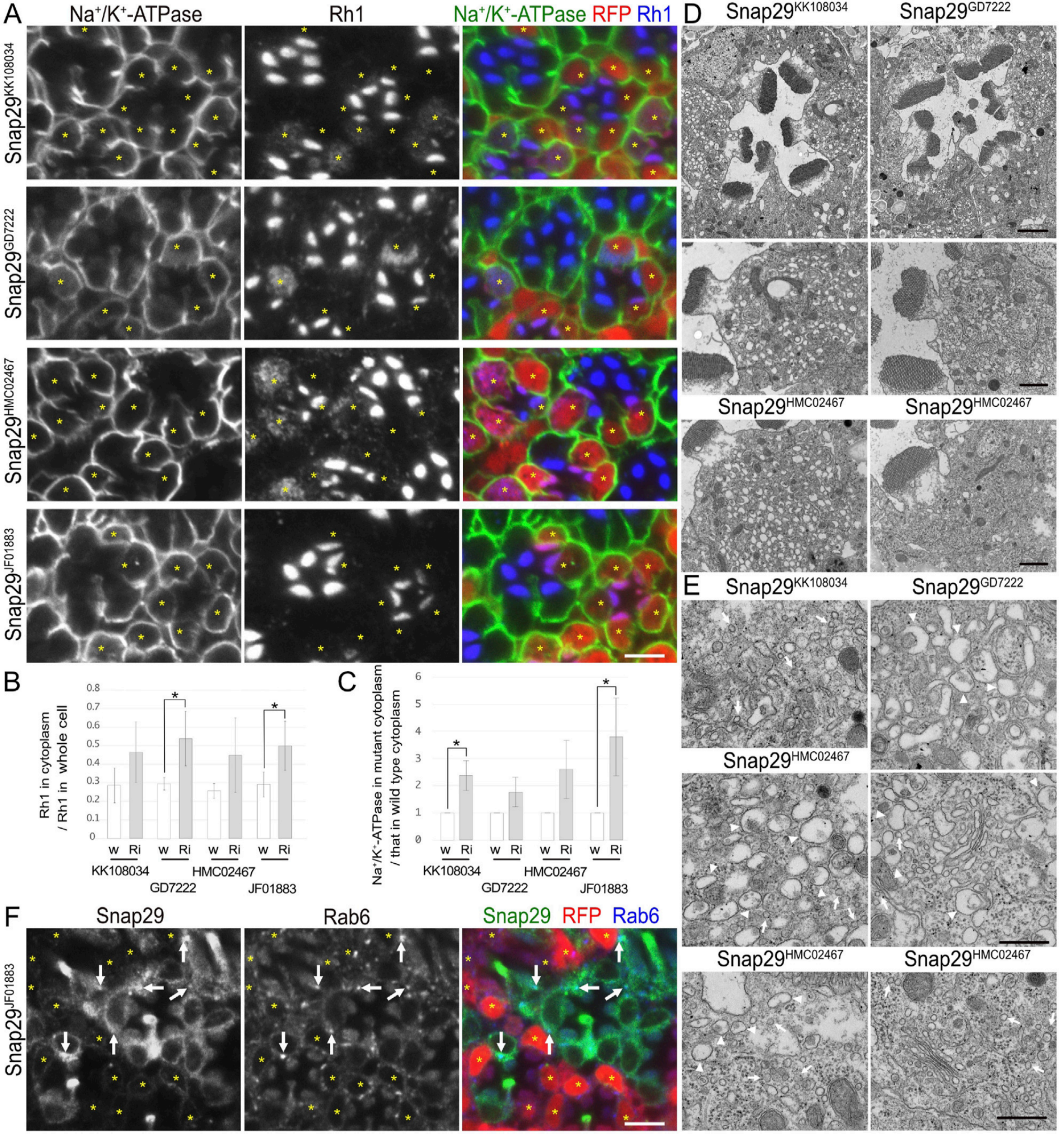
Snap29 is required for the polarized transports toward both the rhabdomeres and basolateral membrane. **(A)** Immunostaining of Snap29RNAi^KK108034^-, Snap29RNAi^GD7222^-, Snap29RNAi^HMC02467^-, or Snap29RNAi^JF01883^-expressing retina by eyeless–CoinFLP–longGMR–Gal4 using anti-Na^+^/K^+^-ATPase-α (green) and anti-Rh1 (blue) antibodies. RFP (red) and asterisks represent the cells expressing RNAi constructs. **(B)** The ratio of integrated fluorescence density for Rh1 staining of the cytoplasm compared with that of whole cells was plotted. White bars indicate wild-type cells (w), and gray bars indicate the cells expressing RNAi constructs (Ri). Error bars indicate the SD with four retinas. Significance according to two-tailed unpaired Student’s *t-*test: *p < 0.05. **(C)** The ratio of integrated fluorescence density for Na^+^/K^+^-ATPase-α staining of the cytoplasm in the cells expressing RNAi constructs compared with that in wild-type cells was plotted. White bars indicate wild-type cells (w), and gray bars indicate the cells expressing RNAi constructs (Ri). Error bars indicate the SD with four retinas. Significance according to two-tailed unpaired Student’s *t-*test: *p < 0.05. **(D)** Electron micrographs of the ommatidia or photoreceptors expressing Snap29RNAi. Used RNAi lines are indicated. **(E)** Electron micrographs of the vesicles accumulated in the cytoplasm or Golgi stacks in the cells expressing Snap29RNAi. Used RNAi lines are indicated. Arrowheads and arrows indicate unusual irregular-shaped vesicles and normal vesicles, respectively. **(F)** Immunostaining of the retina expressing Snap29RNAi^JF01883^ using anti-Snap29 (green) and Rab6 (blue) antibodies. RFP (red) and asterisks represent the cells expressing Snap29RNAi^JF01883^. Arrows indicate Snap29 localization on Golgi stacks. Scale bar: 5 μm **(A)**, 2 μm [**(D)** in ommatidia], 1 μm [**(D)** in photoreceptors], 500 nm **(E)**, and 5 μm **(F)**.

**FIGURE 5:**
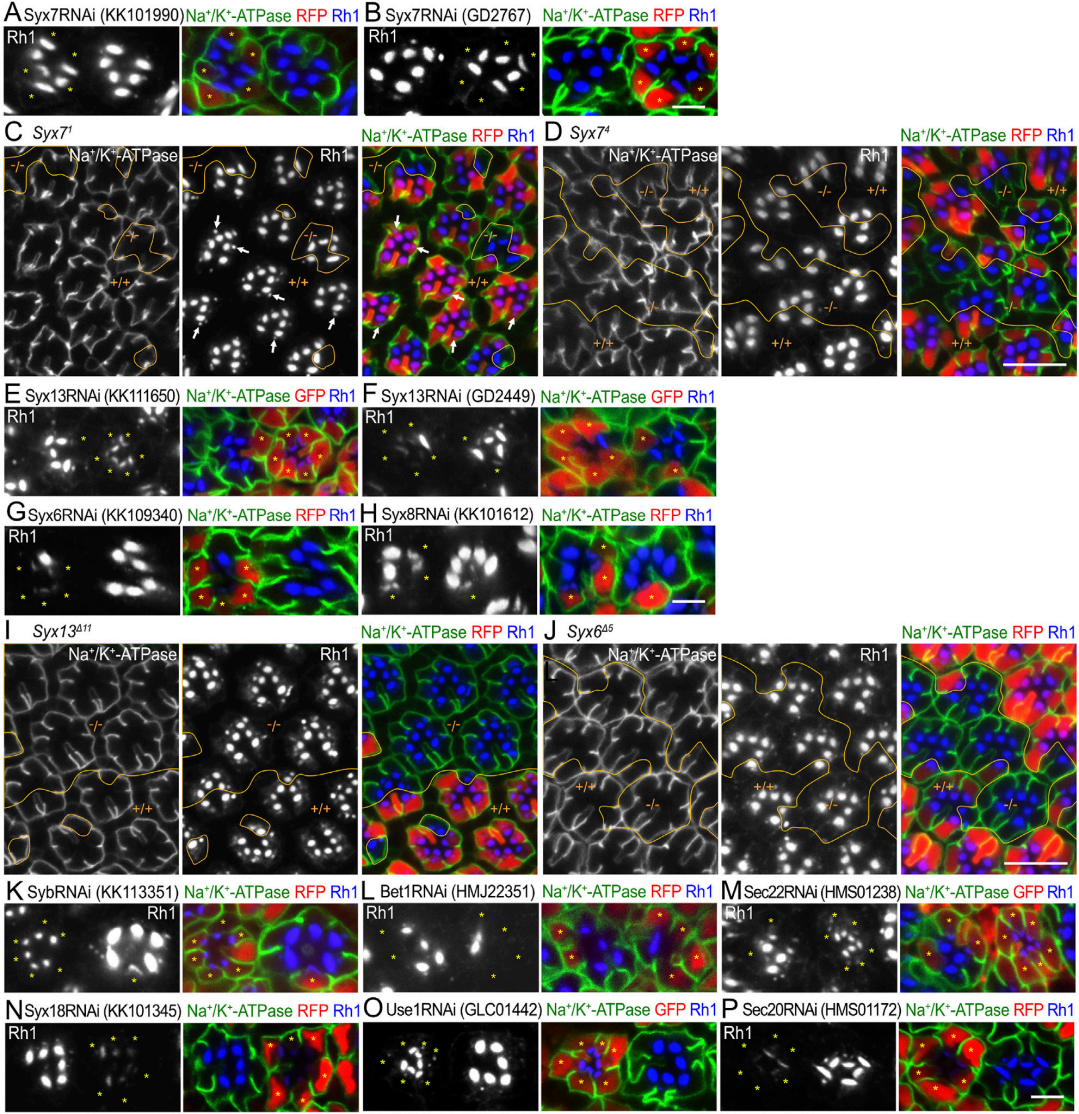
SNARE RNAi mosaic retinas showing category-III phenotypes **(A, B).** Immunostaining of Syx7RNAi^KK101990^ **(A)** or Syx7RNAi^GD2767^ **(B)**-expressing retina by eyeless–CoinFLP–longGMR–Gal4 using anti-Na^+^/K^+^-ATPase-α (green) and anti-Rh1 (blue) antibodies. RFP (red) and asterisks represent the cells expressing RNAi constructs. **(C, D)** Immunostaining of *Syx7* ^*1*^ **(C)** or *Syx7* ^*4*^ **(D)** mosaic retina using anti-Na^+^/K^+^-ATPase-α (green) and anti-Rh1 (blue) antibodies. RFP (red) represents wild-type cells. Arrows indicate Rh1-containing large vesicles. **(E–H)** Immunostaining of SNARE RNAi construct-expressing retina by eyeless–CoinFLP–Act5C–Gal4 **(E, F)** or eyeless–CoinFLP–longGMR–Gal4 **(G, H)** using anti-Na^+^/K^+^-ATPase-α (green) and anti-Rh1 (blue) antibodies. GFP or RFP (red) and asterisks represent the cells expressing RNAi constructs. **(I, J)** Immunostaining of *Syx13* ^*Δ11*^ **(I)** or *Syx6* ^*Δ5*^ **(J)** mosaic retina using anti-Na^+^/K^+^-ATPase-α (green) and anti-Rh1 (blue) antibodies. RFP (red) represents the wild-type cells. **(K–P)** Immunostaining of SNARE RNAi construct-expressing retina by eyeless-CoinFLP–Act5C–Gal4 **(M, O)** or eyeless–CoinFLP–longGMR–Gal4 **(K, L, N, P)** using anti-Na^+^/K^+^-ATPase-α (green) and anti-Rh1 (blue) antibodies. GFP or RFP (red) and asterisks represent the cells expressing RNAi constructs. **(K)** SybRNAi^KK113351^, **(L)** Bet1RNAi^HMJ22351^, **(M)** Sec22RNAi^HMS01238^, **(N)** Syx18RNAi^KK101345^, **(O)** Use1RNAi^GLC01442^, and **(P)** Sec20RNAi^HMS01172^. Scale bar: 5 μm **(A, B)**, 10 μm **(C, D)**, 5 μm **(E–H)**, 10 μm **(I, J)**, and 5 μm **(K–P)**.

Through the three-step mosaic screening of 59 SNARE RNAi lines, we identified 24 lines with meaningful phenotypes (Supplementary Table S1). The lines were classified into three categories (Table 1). Category I represents RNAi lines that induce a strong reduction in Rh1 in the rhabdomeres and accumulate Rh1 in the cytoplasm. This phenotype is typically observed in photoreceptors lacking genes involved in post-Golgi transport to the rhabdomeres, as shown by Rab11RNAi^GD11761^ (Figure 1C) (Li et al., 2007; Otsuka et al., 2019; Satoh et al., 2005; Yamashita et al., 2022). A limited Na^+^/K^+^-ATPase signal of both alpha (α) is also detected in the cytoplasm. Category I included three RNAi lines against nSyb (Figures 2A, D, E) and three against Ykt6 (Figures 2B, F, G). Category II represents RNAi lines that induce not only weak Rh1 and Na^+^/K^+^-ATPase-α cytoplasmic accumulation but also strong Na^+^/K^+^-ATPase mislocalization to the stalk membrane and shortened basolateral membranes. The latter is a characteristic phenotype caused by the loss of genes involved in post-Golgi trafficking to the basolateral membrane, as observed in Rab10RNAi^JF02058^ (Figure 1C). Four RNAi lines targeting Snap29 (Figure 4) and two lines targeting Syx1A (Figure 3) were classified as category II. Category III represents RNAi lines that induce a large reduction in Rh1 in the rhabdomeres without the cytoplasmic accumulation of Rh1. This phenotype is typical of photoreceptors deficient in ER-to-Golgi or intra-Golgi trafficking, as observed in Syx5RNAi^JF03330^ (Figure 1C), but it can also be observed in the defects of genes that regulate other processes. At least one RNAi line for the genes *Syx6*, *Syx7*, *Syx8*, *Syx13*, *Syx18*, *Bet1*, *Use1*, *Sec20*, *Sec22*, and *Syb* was classified as category III (Figure 5).

**TABLE 1:** List of RNAi and gRNA constructs giving the eye phenotypes.

Category I	Category II	Category III	
A SNARE RNAi screening			
nSybJF023417	Syx1A^JF01829^	Syx7^KK101990^	Syx6^KK109340^
nSybGD4553	Syx1A^GD564^	Syx7^GD2767^	Syx8^KK101612^
nSybGD17382	Snap29^JF01883^	Syx13^KK111650^	Bet1^HMJ22351^
Ykt6HMJ21032	Snap29^GD7222^	Syx13^GD2449^	Use1^GLC01442^
Ykt6GD8927	Snap29^KK108034^	Syx18^KK101345^	Sec22^HMS01238^
Ykt6KK101343	Snap29^HMC02467^	Sec20^HMS01172^	Syb^KK113351^
B SNARE gRNA screening			
−	Snap24/25gRNA	Bet1gRNA	Sec22gRNA
	Snap24/25/29gRNA		

(A, B) List of RNAi (A) and gRNA (B) constructs showing the eye phenotypes. Category I represents the RNAi and gRNA lines that induce a strong reduction in Rh1 in the rhabdomeres and instead accumulate Rh1 in the cytoplasm without a significant effect on Na^+^/K^+^-ATPase. Category II represents the RNAi and gRNA lines that induce not only Rh1 cytoplasmic accumulation but also Na^+^/K^+^-ATPase mislocalization to the stalk membrane and shortened basolateral membranes. Category III represents the RNAi and gRNA lines that induce a significant reduction in Rh1 in the rhabdomeres without the accumulation of Rh1 in the cytoplasm.

### 3.4 Two R-SNAREs, nSyb and Ykt6, are strongly involved in post-Golgi transport of Rh1

We recently reported that photoreceptor cells homozygous for null or hypomorphic alleles of nSyb show a defect in Rh1 transport to the rhabdomeres, accompanied by massive cytoplasmic accumulation of Rh1 (Yamashita et al., 2022). As expected, the expression of nSybRNAi^JF023417^, nSybRNAi^GD4553^, or nSybRNAi^GD17382^ reduced Rh1 in the rhabdomeres and strongly induced Rh1 accumulation in the cytoplasm (Figure 2A). In nSyb-knockdown photoreceptors in nSybRNAi^JF023417^ or nSybRNAi^GD17382^ mosaic retinas, 87% ± 7% or 70% ± 13% of Rh1 was localized to the cytoplasm in nSyb-knockdown photoreceptors, whereas only 42% ± 7% or 32% ± 8% of Rh1 was localized in the cytoplasm in control cells in the same retinas (Figure 2C). In contrast to the strong inhibitory effect on Rh1 transport, the basolateral transport of Na^+^/K^+^-ATPase was not severely affected by the expression of nSybRNAi^JF023417^, nSybRNAi^GD4553^, or nSybRNAi^GD17382^. Mislocalization of Na^+^/K^+^-ATPase was not observed, but some Na^+^/K^+^-ATPase α-signals were found in the cytoplasm (Figure 2A). In nSyb-reduced photoreceptors in nSybRNAi^JF023417^ and nSybRNAi^GD17382^ mosaic retinas, cytoplasmic Na^+^/K^+^-ATPase-α was increased to 1.42 ± 0.39-fold and 1.35 ± 0.20-fold, respectively, of that in wild-type cells (Figure 2D), although the significance was achieved only in photoreceptors expressing nSybRNAi^JF023417^. We next investigated the localization of a phototransduction channel in the rhabdomeres, transient receptor potential (TRP), Na^+^/K^+^-ATPase beta subunit (Nrv), inter-rhabdomeric space (IRS)-making protein, Eys, and a stalk membrane protein, Crb. Some TRP and Nrv are accumulated in the cytoplasm, similar to Rh1 and Na^+^/K^+^-ATPase-α in nSyb-reduced photoreceptors (Supplementary Figure S1A left). In nSyb-reduced photoreceptors in nSybRNAi^GD17382^ mosaic retinas, cytoplasmic Nrv was increased to 2.00 ± 1.12-fold of that in wild-type cells (Supplementary Figure S1C), although there was no significance. However, Eys and Crb were normally localized in the IRS and stalk membrane (Supplementary Figure S1A, right). These results indicated that nSyb mainly regulates the transport to the rhabdomeres. Na^+^/K^+^-ATPase accumulation in cytoplasm is surprising, but a normal level of Na^+^/K^+^-ATPase in the basolateral membrane indicates that the role of nSyb in basolateral transport is rather limited compared to the rhabdomere transport.

In addition, the expression of Ykt6RNAi^HMJ21032^, Ykt6RNAi^GD8927^, and Ykt6RNAi^KK101343^ strongly induced Rh1 accumulation in the cytoplasm (Figure 2B). In Ykt6-reduced photoreceptors in Ykt6RNAi^HMJ21032^, Ykt6RNAi^GD8927^, and Ykt6RNAi^KK101343^ mosaic retinas, 84% ± 12%, 85% ± 6%, and 82% ± 9% of Rh1 were localized in the cytoplasm, respectively, whereas only 34% ± 7%, 34% ± 5%, and 30% ± 5% of Rh1 were localized to the cytoplasm in control cells of the same retina, respectively (Figure 2C). These three RNAi lines also induced some cytoplasmic accumulation and minor mislocalization of Na^+^/K^+^-ATPaseα (Figure 2B), although shrinkage of the basolateral membrane was not prominent. In Ykt6-reduced photoreceptors in Ykt6RNAi^HMJ21032^, Ykt6RNAi^GD8927^, and Ykt6RNAi^KK101343^ mosaic retinas, cytoplasmic Na^+^/K^+^-ATPase-α was increased to 1.75 ± 0.57-fold, 1.79 ± 0.54-fold, and 1.73 ± 0.47-fold, respectively, of that in wild-type cells (Figure 4C). Some Nrv, TRP, Eys, and Crb were also accumulated in the cytoplasm (Supplementary Figures S1B, C). In Ykt6-reduced photoreceptors in Ykt6RNAi^KK101343^ and Ykt6RNAi^GD8927^ mosaic retinas, cytoplasmic Nrv was significantly increased to 2.22 ± 0.70-fold and 2.14 ± 0.74-fold, respectively, of that in wild-type cells (Supplementary Figure S1C). Ykt6 might be involved in the post-Golgi transport toward the rhabdomeres, stalk, and basolateral membrane, although the effects on rhabdomere transport were quite severe compared to the other domains. To confirm the involvement of Ykt6 in post-Golgi transport, we attempted to observe the phenotypes of Ykt6 homozygous null clones in the mosaic retina; however, neither *Ykt6* ^*C*^ nor *Ykt6* ^*G0155*^ homozygous clones were obtained, indicating the strong cell lethality of Ykt6 null alleles.

To understand the detailed phenotype of nSyb or Ykt6 RNAi knockdown, thin sections of pupal photoreceptors were examined using electron microscopy (Figures 2E–P). Photoreceptor cells expressing nSybRNAi^JF023417^, nSybRNAi^GD17382^, Ykt6RNAi^GD8927^, and Ykt6RNAi^KK101343^ exhibited smaller rhabdomeres and accumulated cytoplasmic vesicles. These vesicles were irregularly shaped and typically 200 nm in size, which was much larger than normal secretory vesicles (Figures 2H–K, arrowheads). The appearance of these vesicles was similar to that of pleomorphic vesicles that accumulated in the cytoplasm of photoreceptors lacking factors for post-Golgi transport to rhabdomeres, Rab11, Rip11, didum, or exocyst complexes (Beronja et al., 2005; Li et al., 2007; Satoh et al., 2005). Golgi stacks showed normal morphologies in nSyb and Ykt6 knockdown photoreceptors (Figures 2L–P). nSyb localized particularly at the base of the rhabdomeres with Rab11 (Supplementary Figure S1D, arrows) and also Golgi stacks (Yamashita et al., 2022). These results indicate that both nSyb and Ykt6 are required for post-Golgi transport.

### 3.5 Syx1A and Snap29 are involved in post-Golgi transport for both rhabdomeres and basolateral membranes

In addition to the nSyb and Ykt6 RNAi constructs, Syx1A and Snap29RNAi constructs also induced the cytoplasmic accumulation of Rh1, but their effects were rather limited (Figures 3A, 4A). In Syx1A-knockdown photoreceptors in Syx1ARNAi^JF01829^ and Syx1ARNAi^GD564^ mosaic retinas, 49% ± 8% and 54% ± 5% of Rh1 were localized in the cytoplasm, respectively, whereas only 38% ± 8% and 40% ± 10% of Rh1 were localized in the cytoplasm of control cells in the same retinas (Figure 3B). In Snap29-reduced photoreceptors in Snap29RNAi^KK108034^, Snap29RNAi^GD7222^, Snap29RNAi^HMC02467^, and Snap29RNAi^JF01883^ mosaic retinas, 46% ± 16%, 54% ± 15%, 45% ± 20%, and 50% ± 13% of Rh1 were localized in the cytoplasm, respectively, whereas only 29% ± 9%, 29% ± 3%, 26% ± 4%, and 29% ± 7% of Rh1 were localized in the cytoplasm of the control cells in the same retina (Figure 4B).

In addition to Rh1, Na^+^/K^+^-ATPase-α was accumulated in the cytoplasm in Syx1A or Snap29-knockdown photoreceptor cells in similar or severer levels than those in nSyb- or Ykt6-deficient photoreceptors (Figures 3A, 4A). In Syx1A-reduced photoreceptors in Syx1ARNAi^JF01829^ and Syx1ARNAi^GD564^ mosaic retinas, cytoplasmic Na^+^/K^+^-ATPase-α was significantly increased to 1.54 ± 0.32-fold and 1.54 ± 0.22-fold in wild-type cells (Figure 3C). In Snap29-reduced photoreceptors in Snap29RNAi^KK108034^, Snap29RNAi^GD7222^, Snap29RNAi^HMC02467^, and Snap29RNAi^JF01883^ mosaic retinas, cytoplasmic Na^+^/K^+^-ATPase-α was increased to 2.37 ± 0.55-fold, 1.76 ± 0.55-fold, 2.59 ± 1.07-fold, and 3.79 ± 1.43-fold, respectively, of that in wild-type cells (Figure 4C), although the significance was achieved only in photoreceptors expressing Snap29RNAi^KK108034^ and Snap29RNAi^JF01883^. Moreover, the shrinkage of the basolateral membrane was prominent in both Syx1A and Snap29-deficient photoreceptors (Figures 3A, 4A), which is in contrast to nSyb or Ykt6-deficient photoreceptors. Syx1A reduction by Syx1A^JF01829^ expression and Snap29 reduction by Snap29RNAi^JF01883^ or Snap29RNAi^HMC02467^ expression were confirmed by anti-Syx1A or anti-Snap29 antibody staining (Figure 4F; Supplementary Figures S2A, B). Nrv, TRP, Eys, and Crb localizations were also investigated in Snap29RNAi^KK108034^ and Snap29RNAi^GD7222^ mosaic retinas. Only Nrv was accumulated in the cytoplasm, and the localization of TRP, Eys, and Crb was not affected (Supplementary Figures S2C, D). In Snap29-reduced photoreceptors in Snap29RNAi^KK108034^ and Snap29RNAi^GD7222^ mosaic retinas, cytoplasmic Nrv was significantly increased to 1.39 ± 0.27-fold and 1.40 ± 0.25-fold, respectively, of that in wild-type cells (Supplementary Figure S1C).

Similar to nSyb or Ykt6-knockdown photoreceptors, electron microscopy results showed that in photoreceptors expressing either Syx1ARNAi^JF01829^, Syx1ARNAi^GD564^, Snap29RNAi^KK108034^, Snap29RNAi^GD7222^, Snap29RNAi^HMC02467^, or Snap29RNAi^JF01883^, the rhabdomeres were thinner and vesicles accumulated in the cytoplasm (Figures 3D–H, 4D). However, these vesicles were of two types: large pleomorphic vesicles, as observed in nSyb or Ykt6 mutants (Figures 3F–H, 4E, arrowheads), and regularly shaped vesicles with diameters of approximately 100 nm, which are typical secretory vesicles (Figures 3F–H, 4E, arrows). These regular small vesicles were likely post-Golgi vesicles destined for the basolateral membranes. In contrast, the Golgi stacks were unaffected by the reduction in Syx1A or Snap29 in photoreceptors (Figures 3I, 4E; Supplementary Figure S2E).

Next, we examined the localization of endogenous Syx1A and Snap29 using specific antibodies (Figures 3J, 4F). Syx1A appeared to localize exclusively to the rhabdomeres in wild-type cells (Figure 3J), but its basolateral localization was pronounced in EMC3-deficient cells, which have unpacked small rhabdomeres (Satoh et al., 2015). Even if Syx1A is located on both the basolateral and rhabdomere membranes, it may only stain the rhabdomeres because the rhabdomere membranes are too condensed in wild-type cells. Immunostaining of Snap29RNAi^JF01883^ mosaic retina showed an anti-Snap29 antibody in the cytoplasm (Figure 4F) and Golgi stacks (Figure 4F arrows) in the wild-type cells. These results are consistent with the hypothesis that Syx1A and Snap29 are involved in post-Golgi trafficking.

### 3.6 Candidate genes involved in the transport between the ER and Golgi stacks

Category III represents RNAi lines that induce a large reduction in Rh1 in rhabdomeres without ectopic accumulation anywhere in the cell (Figure 5). Two Syx7RNAi constructs, Syx7RNAi^KK101990^ and Syx7RNAi^GD2767^, showed a mild reduction in Rh1 expression and disorganization of rhabdomeres (Figures 5A, B). It is reported that Syx7 is involved in protein degradation in the stalk, rhabdomeres, and basolateral membrane, as well as in the secretion of proteins (Laffafian and Tepass, 2019). As Syx7 null alleles, *Syx7* ^*1*^ and *Syx7* ^*4*^ are available from stock centers but are lethal; we examined the phenotypes of FRT mosaic retinas with wild-type and homozygous photoreceptors of these null mutants. Clones homozygous for *Syx7* ^*1*^ or *Syx7* ^*4*^ were small, and the ommatidia often contained fewer photoreceptors, indicating that *Syx7* ^*1*^ or *Syx7* ^*4*^ alleles were cell-lethal (Figures 5C, D). However, photoreceptors homozygous for either *Syx7* ^*1*^ or *Syx7* ^*4*^ were not severely malformed, although the rhabdomeres of *Syx7* ^*1*^ but not *Syx7* ^*4*^ homozygous photoreceptors were thinner or more disorganized (Figures 5C, D). In contrast, Rh1-containing large vesicles (Figure 5C arrows), supposed to be multivesicular bodies (Satoh et al., 2005; Satoh and Ready, 2005), were not found in *Syx7* ^*1*^ homozygous photoreceptors (Figure 5C), suggesting impaired endocytosis in Syx7 null cells, which is consistent with the results of previous studies (Laffafian and Tepass, 2019). Thus, we excluded the possibility that Syx7 is involved in ER–Golgi trafficking or polarized transport.

Two Syx13RNAi constructs, Syx13RNAi^KK111650^ and Syx13RNAi^GD2449^ (Figures 5E, F); one Syx6RNAi construct, Syx6RNAi^KK109340^ (Figure 5G); and one construct Syx8RNAi^KK101612^ (Figure 5H) showed a strong reduction in Rh1. Since null alleles for Syx13, Syx6, and Syx8 were not available, we attempted to generate them using the CRISPR/Cas9 system. Null alleles of Syx13 and Syx6 were successfully generated; however, we failed to obtain a null allele of Syx8. We examined the phenotypes of photoreceptors homozygous for these null alleles in FRT mosaic retinas. The clones homozygous for either *Syx13* ^*Δ11*^ or *Syx6* ^*Δ5*^ were large but did not show an obvious phenotype (Figures 5I, J). Similarly, one SybRNAi construct, SybRNAi^KK113351^, showed a severe reduction in Rh1 (Figure 5K); however, we previously showed that there is no phenotype for *Syb* ^*21-15*^ or *Syb* ^*25-77*^ (Yamashita et al., 2022). Thus, the RNAi constructs for Syx13, Syx6, and Syb were mistargeted to other genes, and Syx13, Syx6, and Syb were not required for transport between the ER and Golgi stacks or for polarized transport.

Bet1, Syx5, and Sec22 were involved in anterograde trafficking between the ER and Golgi stacks via COPII vesicles. As expected, a strong reduction in Rh1 was observed upon expressing the RNAi construct Bet1RNAi^HMJ22351^ or Sec22RNAi^HMS01238^ (Figures 5L, M). Sec22 reduction by Sec22RNAi^HMS01238^ expression was confirmed by anti-Sec22 antibody staining (Supplementary Figure S2F). Syx18, Use1, Sec20, and Sec22 are SNAREs of Golgi-derived COPI vesicles that fuse with the ER membrane. The expression of RNAi constructs of Syx18^KK101345^, Use1^GLC01442^, or Sec20^HMS01172^ showed strong effects on Rh1 levels (Figures 5N–P). The detailed phenotypes of these SNAREs, which are involved in anterograde and retrograde transport between the ER and Golgi, are described elsewhere (Tago et al., 2024).

### 3.7 CRISPR/Cas9–mosaic eye screening

To expand and complement the mosaic RNAi screening described above, we attempted to utilize the growing resources of transgenic gRNA lines in a scheme involving the instant somatic KO of target genes in the mosaic retina. A project to generate gRNA lines covering every gene in the fly genome is underway using DRSC/TRiP functional genomics resources. In their *in vivo* CRISPR project, fly lines with gRNA designed to knockout genes were called TKO lines. We obtained TKO lines for 15 SNARE genes. Other gRNA lines for Snap24/Snap25/Snap29 were also used in a previous study (Poe et al., 2019). The gRNA lines we used in this study are given in Supplementary Table S2. To generate a mosaic of Cas9 expression in the retina, we generated fly lines carrying constructs of CoinFLP–longGMR–FLAG::Cas9–T2A–Histone2B::2x NeonGreen (H2B::NG) and CoinFLP–Act5C–FLAG::Cas9–T2A–Histone2B::GFP (H2B::GFP). These lines were combined with eyeless-FLP to induce the mosaic expression of FLAG::Cas9 and H2B::NG/GFP. We named them eyeless–CoinFLP–longGMR–FLAG::Cas9-H2B::NG or eyeless–CoinFLP–Act5C-FLAG::Cas9–H2B::GFP, which can generate the somatic mosaic of CRISPR knockout by a single cross with gRNA lines.

**FIGURE 6:**
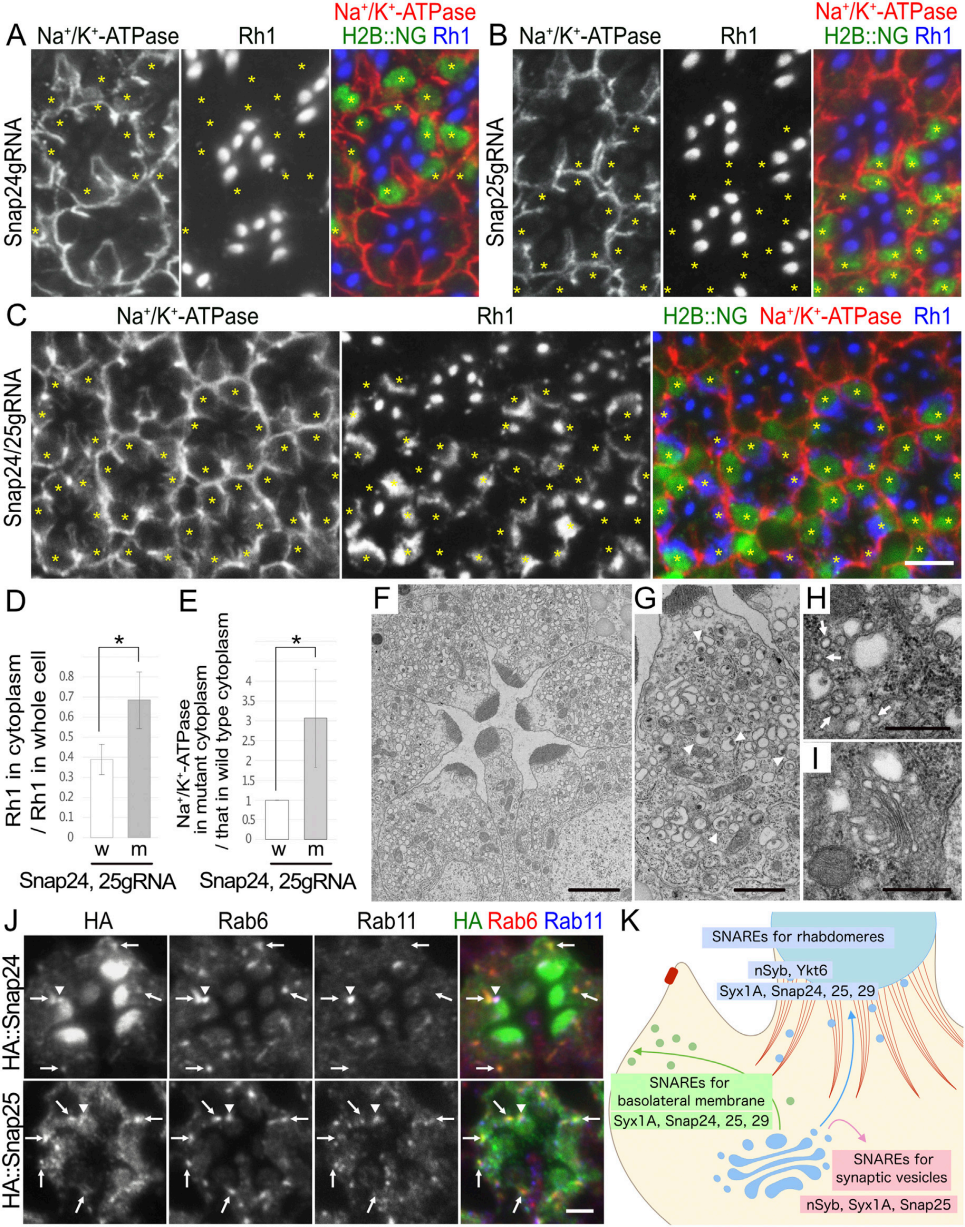
Snap24 and Snap25 are required for the polarized transport toward both the rhabdomeres and basolateral membrane. **(A–C)** Immunostaining of Snap24gRNA **(A)**, Snap25gRNA **(B)**, or Snap24/Snap25gRNA **(C)**-expressing retina combined with eyeless–CoinFLP–longGMR–FLAG::Cas9–H2B::NG using anti-Na^+^/K^+^-ATPase-α (red) and anti-Rh1 (blue) antibodies. H2B::NG (green) and asterisks represents the cells expressing FLAG::Cas9. **(D)** The ratio of integrated fluorescence density for Rh1 staining of the cytoplasm compared with that of whole cells was plotted. White bars indicate wild-type cells (w), and gray bars indicate the cells expressing FLAG::Cas9 (m). Error bars indicate the SD with four retinas. Significance according to two-tailed unpaired Student’s *t-*test: *p < 0.05. **(E)** The ratio of integrated fluorescence density for Na^+^/K^+^-ATPase-α staining of the cytoplasm in the cells expressing FLAG::Cas9 compared with that in wild-type cells was plotted. White bars indicate wild-type cells (w), and gray bars indicate the cells expressing FLAG::Cas9 (m). Error bars indicate the SD with four retinas. Significance according to two-tailed unpaired Student’s *t-*test: *p < 0.05. **(F, G).** Electron micrographs of the ommatidia **(F)** or the photoreceptor **(G)** expressing Poe’s Snap24, Snap25gRNA, and FLAG::Cas9 by eyeless–CoinFLP–longGMR–FLAG::Cas9–H2B::NG. All photoreceptor cells are Snap24/Snap25 double-KO cells. Arrowheads in (**G)** indicate unusual irregular-shaped vesicles. **(H, I)** Electron micrographs of the vesicles accumulated in the cytoplasm **(H)** or Golgi stack **(I)** in the cells expressing Poe’s Snap24, Snap25gRNA, and FLAG::Cas9 by eyeless–CoinFLP–longGMR–FLAG::Cas9–H2B::NG. Arrows in (**H)** indicate normal transport vesicles. **(J)** Immunostaining of the retina expressing HA::Snap24 or HA::Snap25 using anti-HA (green), anti-Rab6 (red), and anti-Rab11 (blue) antibodies. Arrows indicate the dots with both HA and Rab6 staining, and arrowheads indicate the dots with both HA and Rab11 staining. **(K)** Models for SNARE functions on polarized transport. The post-Golgi vesicles for the rhabdomeres and basolateral membrane are shown in blue and green, respectively. F-actin in the retinal terminal web (RTW) and the adherens junction is shown in red. For the post-Golgi transport to the rhabdomere, two R-SNAREs, nSyb and Ykt6, and two Qbc-SNAREs, Snap24/25, are strongly required, and a Qa-SNARE, Syx1A, and a Qbc-SNARE, Snap29, also have some contribution. For the post-Golgi transport to the basolateral membrane, a Qa-SNARE, Syx1A, and a Qbc-SNARE, Snap29, are strongly required, but two R-SNAREs, nSyb and Ykt6, and two Qbc-SNAREs, Snap24/25, also play some roles. An R-SNARE, nSyb, a Qa-SNARE, Syx1A, and a Qbc-SNARE, Snap25, are also involved in the fusion of synaptic vesicles with the synaptic plasma membrane. Scale bar: 5 μm **(A–C)**, 2 μm **(F)**, 1 μm **(G)**, 500 nm **(H, I)**, and 2 μm **(J)**.

Somatic mosaic retinas were generated to test this scheme. We used eyeless-CoinFLP–longGMR–FLAG::Cas9–H2B::NG, which was designed to perform somatic CRISPR KOs in third-instar larvae. Unfortunately, none of the SNARE TKO lines showed any phenotype; however, a previously reported gRNA line expressing both Snap24gRNA and Snap25gRNA (Poe et al., 2019) showed a category-II phenotype: Rh1 accumulation in the cytoplasm and Na^+^/K^+^-ATPase mislocalization to the stalk membrane when these two gRNAs were co-activated by eyeless–CoinFLP–longGMR–FLAG::Cas9-H2B::NG. In contrast, when Snap24gRNA and Snap25gRNA were activated independently, no phenotype was observed in the Cas9-positive clones (Figures 6A–C). In triple-KO photoreceptors of Snap24, Snap25, and Snap29, mosaic retinas contained many Cas9-positive photoreceptors, but none of them showed a category-II phenotype. Instead, the mosaic retinas often contained patches lacking photoreceptors, indicating cell death before the late pupal stage (Supplementary Figure S3A). These results collectively indicate that when CRISPR/Cas9 generates alleles with sufficiently reduced function in all targeted loci, Snap24/Snap25 double-KO photoreceptors are viable, and Snap24/Snap25/Snap29 triple-KO photoreceptors are lethal; however, when one or more of the targeted genes received no or insufficient change by CRISPR/Cas9, the cells could generate photoreceptors in good shape.

Because somatic CRISPR KOs using the longGMR promoter rarely result in phenotypes, we next used the Act5C promoter to perform somatic KOs. The eyeless–CoinFLP–Act5C–FLAG::Cas9–H2B::GFP, in combination with TKO lines, Bet1gRNA, and Sec22gRNA, showed some abnormalities (Supplementary Figures S3B, C). In the Cas9-mosaic retina expressing Bet1gRNA, there were many ommatidia lacking some photoreceptors in the Cas9-positive area, suggesting that some photoreceptor cells died by the late pupal stages, but the surviving cells in this area appeared completely normal (Supplementary Figure S3B). This can be explained if Bet1 is only required at earlier stages of eye development or if CRISPR/Cas9 has created hypomorphic alleles of Bet1 in surviving cells. Similarly, in the Cas9-mosaic retina expressing Sec22gRNA, two types of Cas9-positive cells were found: one showed a severe reduction in Rh1 and the other appeared completely normal (Supplementary Figure S3C). We confirmed the presence of endogenous Sec22 in these retinas by immunostaining with an anti-Sec22 antibody and found that the former cell type lost the Sec22 protein, whereas the latter retained normal levels of the Sec22 protein (Supplementary Figure S3C). Thus, at least in the case of Sec22gRNA, even in Cas9-expressing photoreceptors, some can still express the Sec22 protein at normal levels. In the case of Poe’s gRNA construct, the Act5C-driven Cas9-mosaic retina expressing both Snap24gRNA and Snap25gRNA showed the same abnormality as the longGMR-driven Cas9-mosaic retina; Cas9-expressing cells accumulated Rh1 in the cytoplasm (Supplementary Figure S3D), although the obtained KO clones were small.

### 3.8 Snap24 and Snap25 are involved in post-Golgi transport toward both rhabdomeres and basolateral membranes

We focused on the details of the category-II phenotypes of photoreceptors for probable Snap24/Snap25 double-KO photoreceptors. When double-KO was attempted in third-instar larvae using eyeless–CoinFLP–longGMR–FLAG::Cas9–H2B::NG, virtually all Cas9-positive photoreceptors consistently showed cytoplasmic Rh1 accumulation and Na^+^/K^+^-ATPase-α mislocalization. In Cas9-positive photoreceptors, 68% ± 14% of Rh1 was localized in the cytoplasm, whereas only 39% ± 8% was localized in the cytoplasm of the control cells within the same retinas (Figure 6D). These Snap24/Snap25 double-KO photoreceptors accumulated Na^+^/K^+^-ATPase-α in the cytoplasm: the signal intensity of Na^+^/K^+^-ATPase-α in the cytoplasm was significantly increased to 3.1 ± 1.2-fold that of wild-type cells (Figure 6E; Supplementary Figure S4B). Similar to Na^+^/K^+^-ATPase-α, Nrv was accumulated in the cytoplasm; the signal intensity of Nrv in the cytoplasm was significantly increased to 1.9 ± 0.4-fold that of wild-type cells (Supplementary Figures S4A, B, upper panel). Snap24/Snap25 double-KO photoreceptors also accumulated TRP but not Eys or Crb in the cytoplasm (Supplementary Figure S4A, lower panel).

Furthermore, thin-section electron micrographs revealed that Snap24/Snap25 double-KO photoreceptors had rhabdomeres with shorter microvilli and cytoplasmic vesicle accumulation (Figure 6F). Similar to Syx1A and Snap29, Snap24/Snap25 double-KO photoreceptors accumulated larger irregularly shaped vesicles (Figure 6G, arrowheads) and typical secretory vesicles with a diameter of approximately 100 nm (Figure 6H, arrows). Golgi stacks were not affected by the Snap24/Snap25 double-KO photoreceptors (Figure 6I).

To investigate the localization of Snap24 and Snap25, we generated transgenic flies expressing HA::Snap24 or HA::Snap25. Anti-HA antibody staining of HA::Snap24-expressing ommatidia showed the enrichment of the HA signal in the rhabdomeres and punctate staining in the cytoplasm or at the base of the rhabdomeres (Figure 6J, top panel). Anti-HA antibody staining of HA::Snap25-expressing ommatidia also showed punctate staining in the cytoplasm or at the base of the rhabdomeres but did not show strong signals in the rhabdomeres (Figure 6J, bottom panel). In both HA::Snap24- and HA::Snap25-expressing ommatidia, cytoplasmic dots were colocalized with Rab6 (Figure 6J, arrows), and the dots at the base of the rhabdomeres were colocalized with Rab11 (Figure 6J, arrowheads), indicating that both Snap24 and Snap25 were localized to *trans*-Golgi/TGN and post-Golgi vesicles. It is reasonable to interpret the localization of Snap24 and Snap25 as being involved in post-Golgi trafficking.

## 4 Discussion

In this study, we performed RNAi and somatic CRISPR screening using mosaic eyes to identify SNAREs that regulate the fusion of post-Golgi vesicles to specific membrane domains, rhabdomeres, and basolateral membranes. In addition to nSyb, which we previously reported (Yamashita et al., 2022), we found that the loss of either Ykt6 or Snap24/Snap25 strongly impairs transport to the rhabdomeres. Loss of Syx1A or Snap29 resulted in mild defects in post-Golgi transport toward the rhabdomeres but strong defects in post-Golgi transport toward the basolateral membrane (Figure 6K). Surprisingly, despite extensive screening, no SNARE specifically involved in transport solely to the rhabdomere or the basolateral membrane was identified. Syx1A, Snap25, and nSyb constitute a well-known set of SNAREs that are involved in the fusion of synaptic vesicles to the synaptic plasma membrane (Jahn and Fasshauer, 2012; Kidokoro, 2003; Rao et al., 2001; Schulze et al., 1995; Yoshihara et al., 1999). When overexpressed, Snap24 and nSyb compensate for the synaptic functions of Snap25 and nSyb, respectively (Bhattacharya et al., 2002; Vilinsky et al., 2002). Thus, Syx1A, Snap24, and Snap25 appear to be involved in the fusion of post-Golgi vesicles with all the plasma membrane domains.

Our results are largely consistent with those of a previous RNAi screening of SNAREs performed in non-polarized *Drosophila* S2 cells (Gordon et al., 2017). They identified three sets of SNARE complexes involved in post-Golgi transport (Syx1A, Snap24/Snap29, and Syb; Syx1A, Snap24/Snap29, and Ykt6; and Syx4, Snap24, and Syb) as SNAREs required for the fusion of constitutive secretory carriers with the plasma membrane. The biggest difference and achievement of our work is the identification of neuron-specific SNAREs, nSyb and Snap25, as post-Golgi SNAREs in photoreceptors, because nSyb and Snap25 are expressed in photoreceptor cells but not in S2 cells. Another difference between photoreceptor screening and S2 cell screening is the failure to observe the Syb and Syx4 phenotypes. The former could be explained by the compensation of Syb loss by nSyb, which is expressed only in neurons. The reason for the failure to detect the Syx4 phenotype is not clear; all four Syx4 RNAi lines used in this study might not be strong enough to reduce Syx4, or there might be another SNARE protein that can compensate for Syx4 in photoreceptors but not in S2 cells. Double knockdown of Syx1 and Syx4, but not single knockdown, inhibits collagen secretion in fat cells (Zhou et al., 2023). Thus, the redundancy of SNAREs depends on the tissue or cell. Future studies using Syx4 null alleles or combinatorial RNAi will determine the role of Syx4 in photoreceptors.

The specificity of fusion between transport vesicles and the target membrane is at least partly regulated by the pairing of SNAREs on vesicles (v-SNAREs) and those on the target membrane (t-SNAREs) (Chen and Scheller, 2001; Hong and Lev, 2014). However, surprisingly, we found that none of the SNAREs are solely involved in the post-Golgi transport to the specific plasma membrane domains. However, there seems to be some preferences for each SNARE in regulating the target membrane; nSyb, Ykt6, Snap24, and Snap25 have more functions on the rhabdomeres than on the basolateral membrane, whereas Syx1A and Snap29 seem to have more influence on the basolateral membrane than on the rhabdomeres. Moreover, Syx1A, the only Qa-SNARE identified on the plasma membrane, distributes ubiquitously, and it is also required for post-Golgi transport destined to all plasma membrane subdomains. The fact that both basolateral and rhabdomere transport pathways require the same sets of Qa- and Qbc-SNAREs—Syx1A and Snap24/Snap25/Snap29—suggests that target specificity is determined by factors other than these SNAREs, rather than solely by the combination of R- and Qabc-SNAREs. Although identifying the precise combinations of SNARE complexes required for the specification of membrane subdomains is necessary, our results suggest that in post-Golgi transport to the plasma membrane, SNAREs may not be the sole determinants for vesicles to specify their target subdomains. In fly photoreceptors, the specificity between transport vesicles and target membranes can be controlled by other sets of proteins, such as Rab proteins and tethering factors, rather than SNAREs. Rab11 and Rab35 are required for rhabdomere transport, whereas Rab10 is required for basolateral transport. Such ambiguous situations around the determinant of targeting specificity in fly photoreceptors are consistent with the recent work using proteoliposome microinjection to mammalian cells; liposomes loaded with early or late endosomal SNAREs could be largely targeted to the corresponding endogenous compartments, but their specificity is refined by phosphoinositides and Rab proteins (Koike and Jahn, 2019; Koike and Jahn, 2022; Koike and Jahn, 2024). With the sharp contrast to the post-Golgi SNAREs, the phenotypes observed for ER–Golgi and post-Golgi SNAREs were completely distinct, suggesting that these two groups of SNAREs are not interchangeable. It is possible that the membrane properties between the ER–Golgi and TGN–endosomes are more critically different as they utilize separate ARFGEFs: GBF1 versus BIG1/2 in mammalian cells and Garz versus Sec71 in *Drosophila*.

In this study, we expanded the use of the previously reported CoinFLP. The CoinFLP system, which produces mosaic tissues, is advantageous because it not only overcomes the lethality of mutant flies but also allows for the sophisticated analysis of mutant phenotypes by a side-by-side comparison of wild-type and mutant cells. We added a new promoter system to delay the induction of the phenotype and a somatic CRISPR/Cas9 system using two types of promoters to drive RNAi or Cas9 expression in CoinFLP. The Act5C promoter induces ubiquitous expression, causing Gal4 or Cas9 expression to begin immediately after the CoinFLP system determines whether the cells induce its expression. In contrast, the longGMR promoter delays Gal4 and Cas9 expression until the morphogenetic furrow is reached. This difference in timing allows for cell survival or induction of cell phenotypes. Although the CoinFLP system offers significant advantages for RNAi or gene expression studies, caution should be exercised when used in combination with the somatic CRISPR/Cas9 system. CRISPR/Cas9 induces double-stranded breaks in the target gene; however, the strength of the generated alleles depends on how the chromosomes were repaired in each cell. In case the target gene is essential to survive and grow, the mutant clones in the mosaic retina can be dominated by the escapers carrying weak hypomorphic alleles of the target gene. Consequently, gene functions occasionally appear to be maintained even in Cas9-expressing cells, especially in cases where the loss of function of the gene is highly lethal.

## Data Availability

The original contributions presented in the study are included in the article/Supplementary Material; further inquiries can be directed to the corresponding authors.
