# Supplementary material for: Comprehensive study of SNAREs involved in the post-Golgi transport in Drosophila photoreceptors
Source: Front Cell Dev Biol. 2024 Dec 10;12:1442192. doi: 10.3389/fcell.2024.1442192 (PMC11666571; doi:10.3389/fcell.2024.1442192)
Supplement: Supplementary file 1 [file Presentation1.pdf]

Supplementary Material

Supplemental Material and Methods.

Construction of plasmids

pU62-wsh1

pU62-wsh1 is a plasmid carrying a cassette that works as a "dominant white-eye" marker in Drosophila, consist of Drosophila U6-2 promoter and shRNA against white gene. This vector was constructed based on a vector, pAc-sgRNA-Cas9 (Bassett et al., 2014) by inserting a synthetic shRNA unit against white gene, HMS00017, predesigned in TRiP RNAi & CRISPR fly project (Zirin et al., 2020). Four oligonucleotides TTC-wsh1-F1, wsh1-R1p, wsh1-F2p and wsh1-Ap-GTT were ligated into pAc-sgRNA-Cas9 digested with BbsI, to obtain pU62-wsh1.

pBIDwsh-UASC

pBIDwsh-UASC is a phi31 transgenic vector based on pBID-UASC (Wang et al., 2012) but carrying "dominant white-eye" marker instead of miniature-white “red-eye” marker. From pU62-wsh1, U6-wshRNA cassette was amplified with primers Nh-U62 and gCas9puro-F, then digested with NheI and ApaI, and then cloned between NheI and ApaI sites of pBID-UASC to obtain pBID-wsh-UASC.

pBIDwsh-Act5C-coinFLP-Gal4

pBIDwsh-Act5C-coinFLP-Gal4 is a phi31 transgenic vector carrying "dominant white-eye" marker and coinFLP-Gal4 cassette under control of Act5C promoter. From original coinFLP vector, pAct5C-FRT-stop-FRT3-FRT-FRT3-Gal4 attB (Bosch et al., 2015), the DNA fragment including Act5C promoter and FRT-stop-FRT3-FRT-FRT3-Gal4 cassette was amplified with primers Sbf-BID-Act5C-F and Gal4-BID-Xh-R. Using Gibson assembly, the fragment was cloned between SbfI and BglII sites of pBIDwsh-UASC, replacing UASC promoter and multiple cloning sites, to obtain pBIDwsh-Act5C-coinFLP-Gal4.

5x longGMR promoter

The longGMR promoter (Wernet et al., 2003) consists of five copies of enhancer sequence in the upstream of Rh1 promoter and a basal promoter core of hsp70. Since the detail of the construct was not described, the region containing longGMR promoter was amplified using PCR with primers wdelR_Nhe and Gal4DBD-R2 from a transgenic fly caring P{longGMR-Gal4} (Bloomington Drosophila Stock Center #8605). The sequence of the entire longGMR promoter is available in Dryad.

pP-longGMR, pBID-longGMR and pBIDwsh-longGMR

The fragment containing longGMR promoter was digested with SphI and EcoRI and then ligated between SphI and EcoRI sites of pPdM-UAST (Yamashita et al., 2022), pBID-UASC, and pBIDwsh-UASC, to obtain pP-longGMR, pBID-longGMR, and pBIDwsh-longGMR, respectively.

pP-3xlongGMR, pBID-3xlongGMR and pBIDwsh-3xlongGMR

DNA fragments with various number of LGMR repeats were amplified from pP-longGMR, with primers Sph-longGMR and UAST-R4. The fragments were digested with SphI and EcoRI, then separated by size in agarose gel electrophoresis, and then ligated into pPdM-UAST, pBID-UASC, and pBIDwsh-UASC, to obtain pP-3xlongGMR, pBID-3xlongGMR, and pBIDwsh-3xlongGMR, respectively.

pP-longGMR-coinFLP-Gal4 and pBIDwsh-longGMR-coinFLP-Gal4

From pBIDwsh-Act5C-coinFLP-Gal4, DNA fragments including FRT-stop-FRT3-FRT-FRT3-Gal4 cassette were excised with BamHI and KpnI, and then ligated between BglII and KpnI sites in pP-longGMR, pP-3xlongGMR and pBIDwsh-longGMR to obtain pP-LongGMR-coinFLP-Gal4, pP-3xlongGMR-coinFLP-Gal4 and pBIDwsh-longGMR-coinFLP-Gal4.

pX-HFCas9

A mammalian CRISPR-Cas9 KO vector, pX-HFCas9, coding HiFiCas9^{R691A} (Vakulskas et al., 2018) was constructed based on pSpCas9 (BB)-2A-Puro PX459 V2.0 (Ran et al., 2013), by introducing R691A mutation in the Cas9 gene using Gibson assembly with primers HF-Cas9-F and HF-Cas9-R.

CoinFLP-longGMR-FLAG::Cas9-H2B::2xNG and CoinFLP-Act5C-FLAG::Cas9-H2B::GFP

DNA fragments including HFCas9-T2A was amplified from pX-HF-Cas9 with Bg-Coin-Cas9 and ddT2A-R. DNA fragments including H2B-GFP was amplified from pcDNA3.1 miniSOG2 T2A H2B-EGFP (Makhijani et al., 2017) with ddT2A-F2 and H2B-EGFP-BID-K. Using Gibson assembly, fragments HFCas9-T2A and H2B-2xmNeonGreen were cloned between BglII and KpnI sites of pBIDwsh-Act5C -coin-Gal4 and pP-3xlongGMR-coinFLP-Gal4 to obtain pBIDwsh-Act5C-coinFLP-HFCas9-H2B-GFP and pP-3xlongGMR-coinFLP-HFCas9-H2B-2xNG.

pCFD4w-Syx6

A phi31 vector pCFD4w was constructed by replacing promoter-gRNA scaffold of pCFD5w, a phi31 vector with white+ marker (gift from Michael Boutros, Addgene plasmid #112645), by those of pCFD4. DNA fragments including dU6:2-BbsI-gRNA scaffold were amplified with primers Sgf-U61F and dU6-wsh-fix and digested with EcoRI and NheI, then inserted between EcoRI and XbaI sites of pCFD5w. To construct pCFD4w-Syx6, oligonucleotides Syx6-gRNA3f and Syx6-gRNA3r were annealed and ligated with BbsI digested pCFD4w.

UAS-Myc::Ykt6, UAS-HA::Snap24 and UAS-HA::Snap25

From third instar larvae cDNA, DNA fragment encoding Ykt6 were amplified using primers Xh-dYkt6 and dYkt6-Mlu, then digested with XhoI and MluI, and then ligated between XhoI and MluI sites in pMT-HA-NBgA-m, to generate pMT-HA-Ykt6. The fragment containing Ykt6 was excised with XhoI and XbaI and then inserted into pPdM-UAST-m2NBgAm to generate pUAST-myc-Ykt6. DNA fragment encoding Snap24 and Snap25 were amplified using primers Xh-Snap24, Snap24-Ap, Xh-Snap25 and Snap25-Ap, digested with XhoI and ApaI, and cloned into pMT-HA-NBgA-m to obtain pMT-HA-Snap24 and pMT-HA-Snap25. HA-Snap24 and HA-Snap25 fragments were excised with KpnI and MluI, and then inserted into pPdM-UAST-m2NBgAm to generate pUAST-HA-Snap24 and pUAST-HA-Snap25.

Supplemental references.

Bassett, A. R., Tibbit, C., Ponting, C. P. and Liu, J. L. (2014). Mutagenesis and homologous recombination in Drosophila cell lines using CRISPR/Cas9. *Biol Open* **3** (1), 42-9.

Bosch, J. A., Tran, N. H. and Hariharan, I. K. (2015). CoinFLP: a system for efficient mosaic screening and for visualizing clonal boundaries in Drosophila. *Development* **142** (3), 597-606.

Makhijani, K., To, T. L., Ruiz-González, R., Lafaye, C., Royant, A. and Shu, X. (2017). Precision Optogenetic Tool for Selective Single- and Multiple-Cell Ablation in a Live Animal Model System. *Cell Chem Biol* **24** (1), 110-119.

Ran, F. A., Hsu, P. D., Wright, J., Agarwala, V., Scott, D. A. and Zhang, F. (2013). Genome engineering using the CRISPR-Cas9 system. *Nat Protoc* **8**, 2281-2308.

Vakulskas, C. A., Dever, D. P., Rettig, G. R., Turk, R., Jacobi, A. M., Collingwood, M. A., Bode, N. M., McNeill, M. S., Yan, S., Camarena, J. et al. (2018). A high-fidelity Cas9 mutant delivered as a ribonucleoprotein complex enables efficient gene editing in human hematopoietic stem and progenitor cells. *Nat Med* **24** (8), 1216-1224.

Wang, J. W., Beck, E. S. and McCabe, B. D. (2012). A modular toolset for recombination transgenesis and neurogenetic analysis of Drosophila. *PLoS One* **7** (7), e42102.

Wernet, M. F., Labhart, T., Baumann, F., Mazzoni, E. O., Pichaud, F. and Desplan, C. (2003). Homothorax switches function of Drosophila photoreceptors from color to polarized light sensors. *Cell* **115** (3), 267-79.

Yamashita, H., Ochi, Y., Yamada, Y., Sasaki, S., Tago, T., Satoh, T. and Satoh, A. K. (2022). Functions of neuronal Synaptobrevin in the post-Golgi transport of Rhodopsin in Drosophila photoreceptors. *J Cell Sci* **135** (24), jcs260196.

Zirin, J., Hu, Y., Liu, L., Yang-Zhou, D., Colbeth, R., Yan, D., Ewen-Campen, B., Tao, R., Vogt, E., VanNest, S. et al. (2020). Large-Scale Transgenic. *Genetics* **214** (4), 755-767.

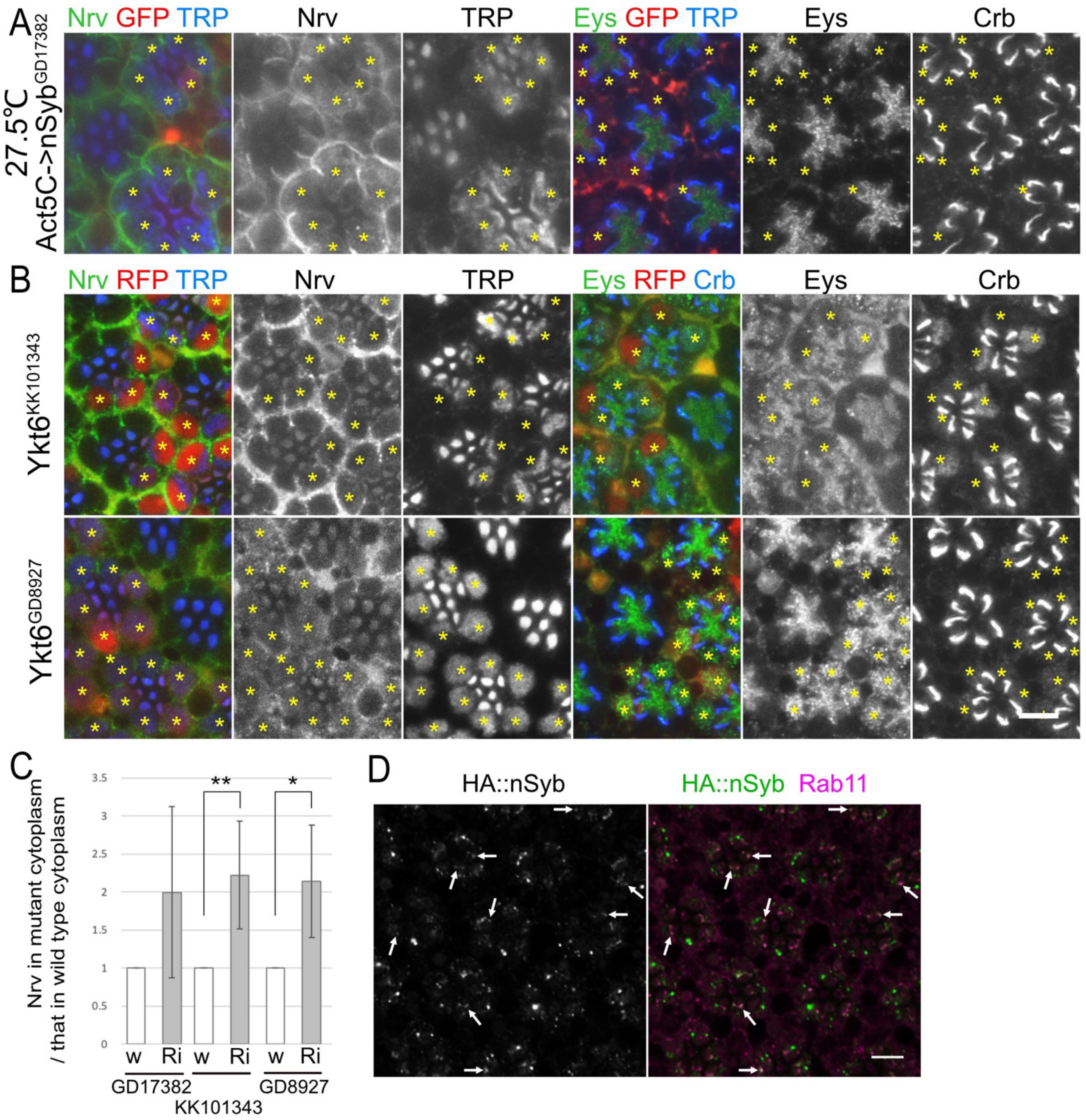
A
27.5°C
Act5C->nSyb^GD17382
Nrv GFP TRP
Nrv
TRP
Eys GFP TRP
Eys
Crb
B
Nrv RFP TRP
Nrv
TRP
Eys RFP Crb
Eys
Crb
Ykt6^KK101343
Ykt6^GD8927
C
Nrv in mutant cytoplasm / that in wild type cytoplasm
0
0.5
1
1.5
2
2.5
3
3.5
**
*
w
Ri
w
Ri
w
Ri
GD17382
KK101343
GD8927
D
HA::nSyb
HA::nSyb Rab11

Supplemental Figure 1. nSyb and Ykt6 are required for the post-Golgi transport.

(A) Immunostaining of nSybRNAi^{GD17382}-expressing retinas by eyeless-CoinFLP-Act5C-Gal4 using anti-Nrv (green) and anti-TRP (blue) antibodies in left, and anti-Eys (green) and anti-Crb (blue) antibodies in right. GFP (red) and asterisks represent the cells

(B) Immunostaining of Ykt6RNAi^{KK101343} or Ykt6RNAi^{GD8927} -expressing retinas by eyeless-CoinFLP-longGMR-Gal4 using anti-Nrv (green) and anti-TRP (blue) antibodies in left, and anti-Eys (green) and anti-Crb (blue) antibodies in right. RFP (red) and asterisks represent the cells expressing RNAi constructs.

C) The ratio of integrated fluorescence density for Nrv staining of the cytoplasm in the cells expressing RNAi constructs compared with that in wild-type cells was plotted. White bars indicate wild-type cells (w) and gray bars indicate the cells expressing RNAi constructs (Ri). Error bars indicate the SD with four retinas. Significance according to two-tailed unpaired Student's *t*-test: $*p < 0.05$, $**p < 0.01$.

(D) Immunostaining of HA::nSyb-expressing retina using anti-HA (green) and anti-Rab11 (magenta) antibodies. Arrows show colocalization of HA::nSyb and Rab11 at the base of the rhabdomeres.

Scale bar: 5 μm (A, B and D).

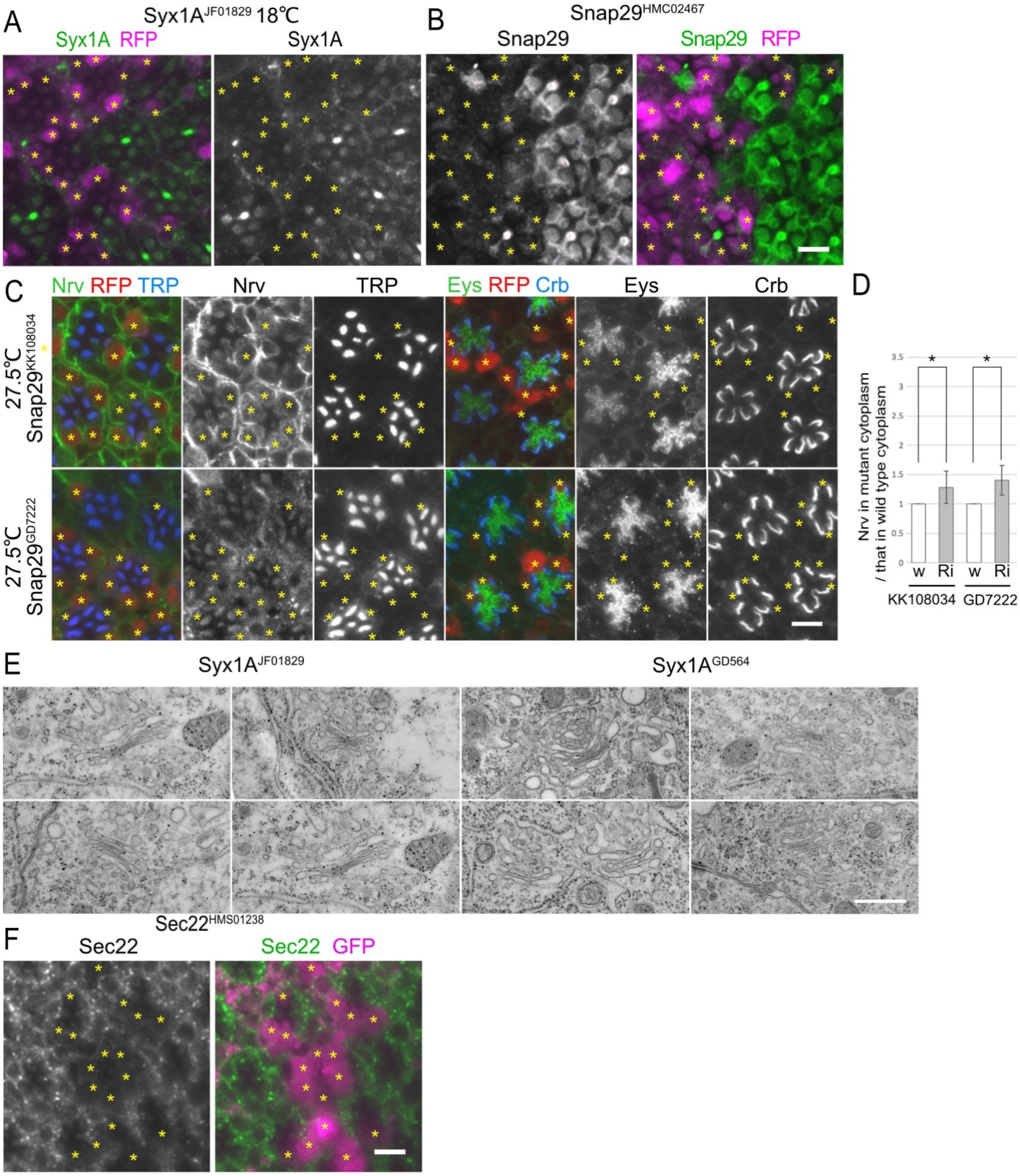
A
Syx1A^JF01829 18℃
Syx1A RFP
Syx1A
B
Snap29^HMC02467
Snap29
Snap29 RFP
C
Nrv RFP TRP
Nrv
TRP
Eys RFP Crb
Eys
Crb
27.5℃ Snap29^KK108034
27.5℃ Snap29^GD7222
D
Nrv in mutant cytoplasm / that in wild type cytoplasm
w
Ri
KK108034
GD7222
E
Syx1A^JF01829
Syx1A^GD564
F
Sec22^HMS01238
Sec22
Sec22 GFP

Supplemental Figure 2. Snap29 is required for the post-Golgi transport.

(A) Immunostaining of Syx1ARNAi^{JF01829}-expressing retina by eyeless-CoinFLP-longGMR-Gal4 using anti-Syx1A (green) antibody. RFP (magenta) and asterisks represents the cells expressing RNAi constructs.

(B) Immunostaining of Snap29RNAi^{HMC02467}-expressing retina by eyeless-CoinFLP-longGMR-Gal4 using anti-Snap29 (green) antibody. RFP (magenta) represents and asterisks the cells expressing RNAi constructs.

(C) Immunostaining of Snap29RNAi^{KK108034} or Snap29RNAi^{GD7222} -expressing retinas by eyeless-CoinFLP-longGMR-Gal4 using anti-Nrv (green) and anti-TRP (blue) antibodies in left, and anti-Eys (green) and anti-Crb (blue) antibodies in right. RFP (red) and asterisks represent the cells expressing RNAi constructs.

(D) The ratio of integrated fluorescence density for Nrv staining of the cytoplasm in the cells expressing RNAi constructs compared with that in wild-type cells was plotted. White bars indicate wild-type cells (w) and gray bars indicate the cells expressing RNAi constructs (Ri). Error bars indicate the SD with four retinas. Significance according to two-tailed unpaired Student's *t*-test: $*p < 0.05$.

(E) Electron micrographs of Golgi stacks in the photoreceptors expressing Syx1ARNAi^{JF01829} (left) and Syx1ARNAi^{GD564} (right).

(F) Immunostaining of Sec22RNAi^{HMS01238}-expressing retina by eyeless-CoinFLP-Act5C-Gal4 using anti-Sec22 (green) antibody. GFP (magenta) and asterisks represents the cells expressing RNAi constructs.

Scale bar: 5 µm (A–C), 500nm (D) and 5 µm (E).

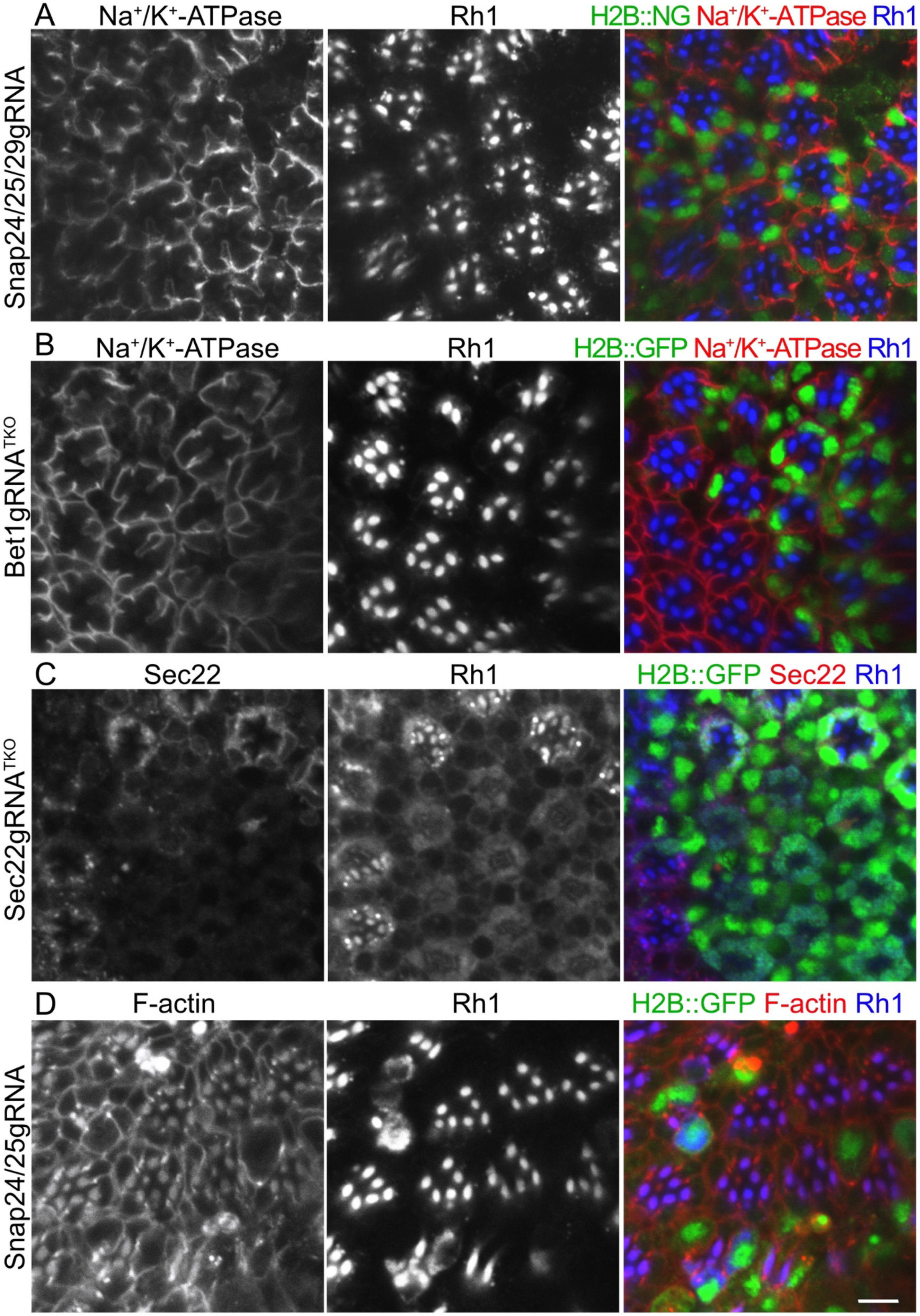

A
Na+/K+-ATPase
Rh1
H2B::NG Na+/K+-ATPase Rh1
Snap24/25/29gRNA
B
Na+/K+-ATPase
Rh1
H2B::GFP Na+/K+-ATPase Rh1
Bet1gRNATKO
C
Sec22
Rh1
H2B::GFP Sec22 Rh1
Sec22gRNATKO
D
F-actin
Rh1
H2B::GFP F-actin Rh1
Snap24/25gRNA

Supplemental Figure 3. gRNA lines show some phenotypes with somatic CRISPR/Cas9.

(A) Immunostaining of Snap24, Snap25 and Snap29gRNA-expressing retina combined with eyeless-CoinFLP-longGMR-FLAG::Cas9-H2B::NG using anti-Na^+/K^+-ATPase-α (red) and anti-Rh1 (blue) antibodies. H2B::NG (green) represents cells expressing FLAG::Cas9.

(B–D) Immunostaining of Bet1gRNA (B) -, Sec22gRNA (C) -, or Snap24/Snap25gRNA (D) -expressing retina combined with eyeless-CoinFLP-Act5C-FLAG::Cas9-H2B::GFP using anti-Na^+/K^+-ATPase-α (red) and anti-Rh1 (blue) antibodies (B), anti-Sec22 (red) and anti-Rh1 (blue) antibodies (C) or phalloidin (red) and anti-Rh1 (blue) antibodies (D). H2B::GFP (green) represents cells expressing FLAG::Cas9.

Scale bar: 5 μm (A–D).

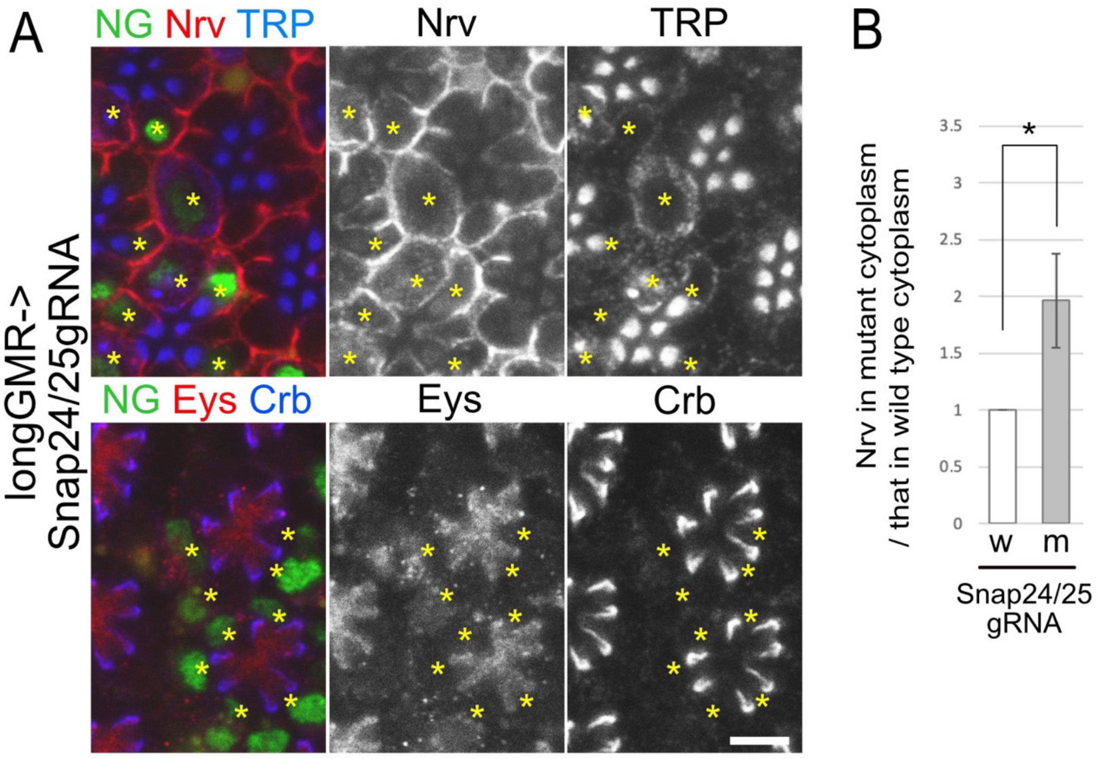

Supplemental Figure 4. Snap24 and Snap25 are required for the post-Golgi transport.

(A) Immunostaining of Snap24/Snap25gRNA-expressing retina combined with eyeless-CoinFLP-longGMR-FLAG::Cas9-H2B::NG using anti-Nrv (red) and anti-TRP (blue) antibodies in upper panel, and anti-Eys (red) and anti-Crb (blue) antibodies in lower panel. NG (green) and asterisks represent the cells expressing gRNA constructs.

(B) The ratio of integrated fluorescence density for Nrv staining of the cytoplasm in the cells expressing RNAi constructs compared with that in wild-type cells was plotted. White bars indicate wild-type cells (w) and gray bars indicate the cells expressing gRNA constructs (m). Error bars indicate the SD with four retinas. Significance according to two-tailed unpaired Student's *t*-test: *$p < 0.05$.

Scale bar: 5 μm (A).

Ochi et al., Table S1

	Gene name	CG #	RNAi line	stock No.	25℃ Act5C-CoinFLPGal4	25℃ longGMR-CoinFLPGal4	20℃ longGMR-CoinFLPGal4
SNARE Qa	Syx1A	CG31136	JF01829	BL25811	no clone	unevaluable	Ⅱ
	Syx1A	CG31136	GD564	v33112	no clone	unevaluable	Ⅱ
	Syx4	CG2715	HMS02771	BL44054	no phenotype		
	Syx4	CG2715	JF01714	BL31198	no phenotype		
	Syx4	CG2715	JF01460	BL31667	no phenotype		
	Syx4	CG2715	GD8646	v32413	no phenotype		
	Syx7	CG5081	KK101990	v107264	no clone	Ⅲ	
	Syx7	CG5081	GD2767	v5413	no clone	Ⅲ	
	Syx13	CG11278	HMS01723	BL38594	no phenotype		
	Syx13	CG11278	JF01920	BL27984	no phenotype		
	Syx13	CG11278	KK111650	v102432	Ⅲ		
	Syx13	CG11278	GD2449	v8361	Ⅲ		
	Syx16	CG1467	JF01924	BL25884	no phenotype		
	Syx16	CG1467	HMC03430	BL51856	no phenotype		
	Syx16	CG1467	KK108039	v109504	no phenotype		
	Syx16	CG1467	GD3694	v8644	no phenotype		
	Syx17	CG7452	JF01937	BL25896	no phenotype		
	Syx17	CG7452	KK100034	v108825	no phenotype		
	Syx17	CG7452	GD14850	v36595	no phenotype		
	Syx18	CG13626	JF02263	BL26721	no phenotype		
	Syx18	CG13626	KK101345	v105113	no clone	Ⅲ	
SNARE Qb	Membrin	CG4780	GLC01633	BL50515	no phenotype		
	Gos28	CG7700	HMS01203	BL34724	no phenotype		
	Vti1a	CG3279	HMS01727	BL38526	no phenotype		
	Vti1a	CG3279	GD2233	v45725	no phenotype		
	Vti1b	CG44009	HMC06083	BL65221	no phenotype		
	Sec20	CG2023	HMS01172	BL34693	no clone	Ⅲ	
SNARE Qc	Syx6	CG7736	JF03125	BL28505	no phenotype		
	Syx6	CG7736	KK109340	v104795	no clone	Ⅲ	
	Syx6	CG7736	GD422	v1578	no phenotype		
	Syx8	CG4109	JF002038	BL26013	no phenotype		
	Syx8	CG4109	KK101612	v107014	unevaluable	Ⅲ	
	Bet1	CG14084	HMS02324	BL41927	no phenotype		
	Bet1	CG14084	HMJ22351	BL58269	no clone	Ⅲ	
	Use1	CG14181	GLC01442	BL43253	Ⅲ		
SNARE Qbc	Snap24	CG9474	JF03146	BL28719	no phenotype		
	Snap24	CG9474	GD4050	v48033	no phenotype		
	Snap25	CG40452	JF02615	BL27306	no phenotype		
	Snap25	CG40452	HMS01367	BL34377	no phenotype		
	Snap29	CG11173	HMC02467	BL51893	no clone	Ⅱ	
	Snap29	CG11173	JF01883	BL25862	no clone	Ⅱ	
	Snap29	CG11173	GD7222	v18172	unevaluable	Ⅱ	
	Snap29	CG11173	KK108034	v107947	unevaluable	Ⅱ	
SNARE R	Syb	CG12210	HMS01987	BL39067	no phenotype		
	Syb	CG12210	HMS02728	BL44014	no phenotype		
	Syb	CG12210	HMS01678	BL38234	no phenotype		
	Syb	CG12210	KK113351	v102922	no clone	Ⅲ	
	Syb	CG12210	GD4534	v30605	no phenotype		
	nSyb	CG17248	JF023417	BL31983	Ⅰ		
	nSyb	CG17248	KK109893	v104531	no phenoype		
	nSyb	CG17248	GD4553	v44011	Ⅰ		
	nSyb	CG17248	GD17382	v49202	Ⅰ		
	Vamp7	CG1599	GL01524	BL43543	no phenotype		
	Vamp7	CG1599	HMS01762	BL38300	no phenotype		
	Ykt6	CG1515	HMS01778	BL38314	no phenotype		
	Ykt6	CG1515	HMJ21032	BL50937	no clone	Ⅰ	
	Ykt6	CG1515	GD8927	v19329	unevaluable	Ⅰ	
	Ykt6	CG1515	KK101343	v105648	no clone	Ⅰ	
	Sec22	CG7359	HMS01238	BL34893	Ⅲ		

Supplemental Table 1. The list of RNAi lines used in this study.

Supplemental Table 2. The list of gRNA lines used in this study.

Ochi et al., Table S2

	Gene name	CG #	gRNA line	stock No.	25℃<br>Act5C-CoinFLPCas9	25℃<br>longGMR-CoinFLPCas9
SNARE Qa	Syx1A	CG31136	BL81800	GS04848	no phenotype	no phenotype
	Syx4	CG2715	BL81462	GS04710	no phenotype	no phenotype
	Syx7	CG5081	BL84033	GS04402	no phenotype	no phenotype
	Syx13	CG11278	BL84021	GS04362	no phenotype	no phenotype
	Syx17	CG7452	BL83543	GS04320	no phenotype	no phenotype
SNARE Qb	Gos28	CG7700	BL84027	GS04369	no phenotype	no phenotype
	Vti1a	CG3279	BL83537	GS04311	no phenotype	no phenotype
	Vti1b	CG44009	BL77221	GS01034	no phenotype	no phenotype
SNARE Qc	Syx6	CG7736	BL84244	GS04342	no phenotype	no phenotype
	Syx8	CG4109	BL83560	GS04349	no phenotype	no phenotype
	Bet1	CG14084	BL83536	GS04309	Ⅲ	no phenotype
SNARE Qbc	Snap24	CG9474	BL84035	GS04409	no phenotype	no phenotype
	Snap25	CG40452	BL80880	GS04784	no phenotype	no phenotype
	Snap29	CG11173	BL83927	GS03522	no phenotype	no phenotype
SNARE R	Sec22	CG7359	BL83805	GS04339	Ⅲ	no phenotype
SNARE Qbc	Snap24	CG9474			no phenotype	no phenotype
	Snap25	CG40452			no phenotype	no phenotype
	Snap29	CG11173			no phenotype	no phenotype
	Snap24/25				Ⅱ	Ⅱ
	Snap24/25/29				Ⅱ (small)	Ⅱ

Ochi et al., Supplemental Table 3

Sequnce Files of Plasmids	Name	Source
pAc-sgRNA-Cas9(49330).gb	pAc-sgRNA-Cas9	Addgene #49330
pAct_FRT_stop_FRT3_FRT_FRT3_Gal4_attB(52889).gb	pAct-FRT-stop-FRT3-FRT-FRT3-Gal4-attB	Addgene #52889
pBID-wsh-coinFLP-Gal4.gb	pBID-wsh-coinFLP-Gal4	This work
pBID-wsh-coinFLP-HFCas9-H2B-EGFP.gb	pBID-wsh-coinFLP-HFCas9-H2B-EGFP	This work
pBID-wsh-coinFLP-HFCas9-H2B-2xNG.gb	pBID-wsh-coinFLP-HFCas9-H2B-2xNG	This work
pBIDwsh-UASC.gb	pBIDwsh-UASC	This work
pCFD4w.gb	pCFD4w	This work
pCFD5.gb	pCFD5	This work
pCFD5w(112645).gb	pCFD5w	Addgene #112645
pMT-HA-NBgA-m.gb	pMT-HA-NBgA-m	This work
pP-3xlongGMR-coinFLP-HFCas9.gb	pP-3xlongGMR-coinFLP-HFCas9	This work
pP-3xlongGMR-coinFLP-HFCas9-H2B-2xNG.gb	pP-3xlongGMR-coinFLP-HFCas9-H2B-2xNG	This work
pP-longGMR-coinFLP-Gal4.gb	pP-longGMR-coinFLP-Gal4	This work
pP-longGMR-coinFLP-HFCas9.gb	pP-longGMR-coinFLP-HFCas9	This work
pP-longGMR.gb	pP-longGMR	This work
pPdm-HA-Snap24.gb	pPdm-HA-Snap24	This work
pPdM-HA-Snap25.gb	pPdM-HA-Snap25	This work
pPdM-UAST-vAX2m.gb	pPdM-UAST-vAX2m	This work
pU62-wsh1.gb	pU62-wsh1	This work
pUAST-Myc-Ykt6.gb	pUAST-Myc-Ykt6	This work
pX-HFCas9.gb	pX-HFCas9	This work
pX459 pSpCas9(BB)-2A-Puro V2_0 (62988).gb	pX459 pSpCas9(BB)-2A-Puro	Addgene #62988
**Other sequence files**		
**Syx13_genomic(11bp-del).gb**<br>Genomic region of Drosophila Syx13, including the genome-edited site. DNA fragments were amplified using primers Syx13-GF1 and Syx13-GR1, then sequenced. The null allele generated in this work, Syx13Δ11(delta-11) has 11 bp deletion shown in red.		
**Syx6-M2-1(5bp-del).gb**<br>Genomic region of Drosophila Syx6, including the genome-edited site. DNA fragments were amplified using primers Syx6-GF3 and Syx6-GR3, then sequenced. The null allele generated in this work, Syx6Δ5(delta-5) has 5 bp deletion shown in red.		
**longGMR_PCR.gb**<br>Sequence of the longGMR promoter (Wernet et al., 2003). The region containing longGMR promoter was amplified using PCR with primers wdelR_Nhe and Gal4DBD-R2 from a transgenic fly caring P{longGMR-Gal4} (Bloomington Drosophila Stock Center #8605).		

Supplemental Table 3. The list of the sequence files dropped to Dryad.

Ochi et al., Supplemental Table 4

Primer name	Sequence
TTC-wsh1-F1	5'-TTCGCAGGAGCTTTCGCTCAGCAAAGTTATAT-3'
wsh1-R1p	5'phos-GCTTGAATATAACTTTGCTGAGCGAAAGCTCCTGC-3'
wsh1-F2p	5'phos-TCAAGCATATTTGCTGAGCGAAAGCTCCTGTTTTTTGGGCCC-3'
wsh1-Ap-GTT	5'-AACGGGCCCAAAAAACAGGAGCTTTCGCTCAGCAAATAT-3'
Nh-U62	5'-ggtggtGCTAGCgttcgacttgcagcctgaaatacg-3'
gCas9puro-F	5'-TGCACCTACTTCTCATTTCCACTGTCAC-3'
Sbf-BID-Act-F	5'-gatccgcttgcatgcctgcaggaTCTCGCTGCCTGTTATGTG-3'
Gal4-BID-Xh-R	5'-aagatccTCTAGAGGTACCTTACTCTTTTTTTGGGTTTGGTGG-3'
wdelR_Nhe	5'-GGGCTAGCTTCAATGATGTCCAGTGCAG-3'
Gal4DBD-R2	5'-GACCTTTTGGTTTTGGGAGAGTAGCG-3'
Sph-LongGMR	5'-gggcatgcgTCGAGacaCCCAGTGGAAACC-3'
UAST-R4	5'-TTAAAGGCATTCCACCACTGCTCCC-3'
HF-Cas9-F	5'-TCGCCAACGCAAACTTCATGCAGCTGATCCACGAC-3'
HF-Cas9-R	5'-CATGAAGTTTGCGTTGGCGAAGCCGTCGGACTT-3'
Bg-Coin-Cas9	5'-gtgtagtgttgcggtgGAATTCAccATGGACTATAAGGACCACGACGGA-3'
ddT2A-R	5'-TGGGCCAGGATTCTCCTCGAC-3'
ddT2A-F2	5'-GGCAGTGGAGAGGGCAGAGGAAgtctgcta-3'
H2B-EGFP-BID-K	5'-cacaaagatccTCTAGAGGTACCTTAAACGGGCCCTCTAGACTTGTA-3'
Xh-dYkt6	5'-GGctcgagTGTCAAACTATTCGCGTTGAGCATC-3'
dYkt6-Mlu	5'-ccACGCGTCTAGGTGAAGCTGCAGCAGGAG-3'
Xh-Snap24	5'-GGCTCGAGTATGGCCGCCGTGGAGAATGCC-3'
Snap24-Ap	5'-ggtGGGCCCTTAACTCTTGAGCAGATTGTTGGCG-3'
Xh-Snap25	5'-GGCTCGAGTatgccagcggatccatctgaa-3'
Snap25-Ap	5'-ggtGGGCCCttactttaatagttgatgtgccctttg-3'
Sgf-U61F	5'-atagccaagaatggaGCGATCGCgaattcattttcaacgtcctcga-3'
dU6-wsh-fix	5'-gaacgctagcAAAAAAGCACCGACTCGGTGCCAC-3'
Syx6-gRNA3f	5'-TTCGTACCATAGCTGGGACCATGT-3'
Syx6-gRNA3r	5'-AAACACATGGTCCCAGCTATGGTA-3'
Syx13-GF1	5'-ttgtctgtttgctcgcgagtggt-3'
Syx13-GR1	5'-gtggaactgcgtgcatttgggtc-3'
Syx6-GF2	5'-atgaaaagaaccgtcaagtgcgcc-3'
Syx6-GR2	5'-gagatcccactcgatgctccgtag-3'
Syx6-GF3	5'-cgatgaggtcaagcagATGAAGG-3'
Syx6-GR3	5'-TGTTTCGCCCACGTACCTATGACT-3'

Supplemental Table 4. The list of the primers we used in this study.
